# Oral administration of bovine milk-derived extracellular vesicles induces senescence in the primary tumor but accelerates cancer metastasis

**DOI:** 10.1038/s41467-021-24273-8

**Published:** 2021-06-24

**Authors:** Monisha Samuel, Pamali Fonseka, Rahul Sanwlani, Lahiru Gangoda, Sing Ho Chee, Shivakumar Keerthikumar, Alex Spurling, Sai V. Chitti, Damien Zanker, Ching-Seng Ang, Ishara Atukorala, Taeyoung Kang, Sanjay Shahi, Akbar L. Marzan, Christina Nedeva, Claire Vennin, Morghan C. Lucas, Lesley Cheng, David Herrmann, Mohashin Pathan, David Chisanga, Sean C. Warren, Kening Zhao, Nidhi Abraham, Sushma Anand, Stephanie Boukouris, Christopher G. Adda, Lanzhou Jiang, Tanmay M. Shekhar, Nikola Baschuk, Christine J. Hawkins, Amelia J. Johnston, Jacqueline Monique Orian, Nicholas J. Hoogenraad, Ivan K. Poon, Andrew F. Hill, Markandeya Jois, Paul Timpson, Belinda S. Parker, Suresh Mathivanan

**Affiliations:** 1 Department of Physiology, Anatomy and Microbiology, School of Life Sciences, La Trobe University, Bundoora, VIC Australia; 2 Department of Biochemistry and Genetics, La Trobe Institute for Molecular Science, La Trobe University, Melbourne, VIC Australia; 3 Cancer Research Division, Peter MacCallum Cancer Centre, Melbourne, VIC Australia; 4 Sir Peter MacCallum Department of Oncology, University of Melbourne, Melbourne, VIC Australia; 5 Bio21 Institute, University of Melbourne, Melbourne, VIC Australia; 6 Garvan Institute of Medical Research, The Kinghorn Cancer Centre & St Vincent’s Clinical School, Faculty of Medicine, University of New South Wales, Sydney, NSW Australia

**Keywords:** Biochemistry, Cancer

## Abstract

The concept that extracellular vesicles (EVs) from the diet can be absorbed by the intestinal tract of the consuming organism, be bioavailable in various organs, and in-turn exert phenotypic changes is highly debatable. Here, we isolate EVs from both raw and commercial bovine milk and characterize them by electron microscopy, nanoparticle tracking analysis, western blotting, quantitative proteomics and small RNA sequencing analysis. Orally administered bovine milk-derived EVs survive the harsh degrading conditions of the gut, in mice, and is subsequently detected in multiple organs. Milk-derived EVs orally administered to mice implanted with colorectal and breast cancer cells reduce the primary tumor burden. Intriguingly, despite the reduction in primary tumor growth, milk-derived EVs accelerate metastasis in breast and pancreatic cancer mouse models. Proteomic and biochemical analysis reveal the induction of senescence and epithelial-to-mesenchymal transition in cancer cells upon treatment with milk-derived EVs. Timing of EV administration is critical as oral administration after resection of the primary tumor reverses the pro-metastatic effects of milk-derived EVs in breast cancer models. Taken together, our study provides context-based and opposing roles of milk-derived EVs as metastasis inducers and suppressors.

## Abstract (web summary)

Dietary extracellular vesicles (EVs) could potentially be absorbed by the intestinal tract of the host and exert multiple phenotypic changes. Here, the authors isolate and characterize EVs from raw and commercial bovine milk and show orally administered EVs to have a context specific role in promoting or suppressing primary tumor growth and metastasis in multiple mouse tumor models.

## Introduction

The idea that ingested dietary RNA may be bioavailable in host organism, and their purported ability to regulate gene expression in tissues and the phenotype of an organism, has ignited unprecedented interest in cross-kingdom and cross-species communication^1,2^. The concept of cross-kingdom communication was further strengthened when high levels of exogenous plant miR159 in human sera negatively correlated with breast cancer incidence and progression^3^. In corroboration with this observation, oral delivery of miR159 significantly reduced xenograft breast tumor burden. Whilst these observations challenged existing paradigms and highlighted that dietary RNAs could be absorbed via food intake, circulate in the blood, reach various organs and modulate gene expression, it also kindled additional debate among the regulatory boards of genetically modified organisms^4,5^. However, the concept was challenged by few well-controlled studies emphasizing the instability of nucleic acids which ultimately succumb to the membrane barriers and nucleases of the mammalian gastrointestinal tract^6,7^. Furthermore, the observations were often criticized as diet-responsive endogenous RNAs or artefacts due to non-adherence of rigorous procedures^5^. While lipid bilayered extracellular vesicles (EVs) are proposed to be protectors of the rich cargo of proteins and RNA^8–11^, concrete in vivo evidence to support cross-species/kingdom communication via EVs are limited and hence the concept remains controversial^12^.

Among mammalian dietary sources, milk is one of the most highly consumed beverages containing an abundance of EVs^8,11,13^. Milk is a uniquely complex epigenetic imprinting system that has evolved to specifically support postnatal growth and has had remarkable impact on human growth and development^14,15^. Being an immensely intricate secretory product, specifically designed for regulation of neonatal development, its persistent consumption throughout the lifespan may have substantial implications on human health^12,16^. Both raw as well as CM have been shown to contain EVs that carry a cargo rich in RNAs and proteins^11,17^. EVs confer protection to the luminal cargo (proteins and RNA) against harsh degrading conditions^18^ and it has recently been demonstrated that milk-derived EVs have been taken up by human and murine gut epithelial cells in vitro^17,19^.

In this study, bovine milk-derived EVs were orally administered to various mouse models, and their role in cross-species communication was assessed. Orally administered milk-derived EVs resisted the harsh environment of the intestinal tract and were bioavailable in a number of mouse organs including the liver. Quantitative proteomics analysis confirmed the presence of bovine-specific proteotypic peptides in liver tissues of mice orally administered with milk-derived EVs. Ingestion of bovine milk-derived EVs reduced the primary tumor growth and attenuated cancer-induced weight loss. Intriguingly, despite reduction in primary tumor size, milk-derived EVs augmented metastasis in various cancer models raising serious implications the diet may have on the fatal steps of this disease. These results highlight the role of milk-derived EVs in cross-species communication and their significant context-dependent role in regulating cancer progression and metastasis.

## Results

### Milk-derived EVs are stable under harsh degrading conditions

**Fig. 1:**
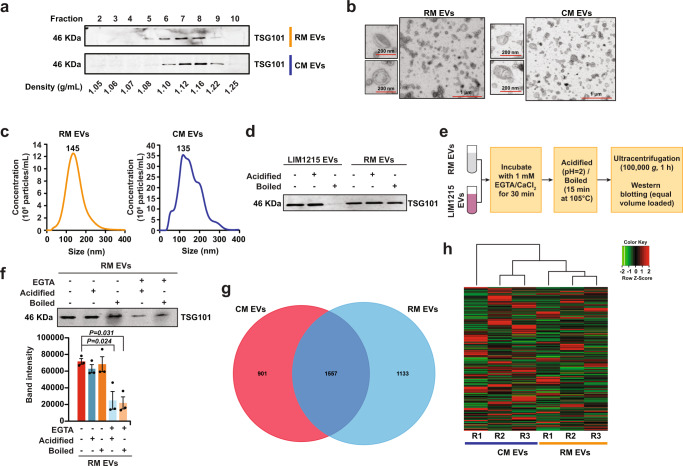
Characterization of bovine milk-derived EVs. **a** Western blot analysis of EV-enriched protein TSG101 in milk-derived EV samples. **b** TEM images of RM and CM EVs. **c** NTA revealed major peak as 145 nm for RM and 135 nm for CM samples. **d** Equal protein amount of EVs from RM and colorectal cancer cells (LIM1215) were acidified (pH = 2) or boiled (15 min at 105 °C), subjected to ultracentrifugation and final total volumes were assessed for stability by western blotting for EV-enriched protein TSG101. **e** Schematic representation of the assay to test the role of calcium in EV stability. **f** Equal amounts of milk-derived EVs were treated with EGTA prior to acidification or boiling. The samples were subjected to ultracentrifugation and final total volumes were assessed by Western blot analysis for TSG101 (*n* = *3*). All data are represented as mean ± s.e.m. Statistical significance was determined by two-tailed *t*-test. **g** Venn diagram of differentially abundant proteins identified in RM and CM EVs (*n* = *3*). **h** Heatmap depicting the small RNA profile of RM and CM EVs (R-biological replicate) (*n* = *3)*.

EVs were isolated from raw milk (RM) and commercial milk (CM) samples by differential centrifugation coupled with ultracentrifugation. The isolated EVs were later separated using OptiPrep density gradient centrifugation. To identify EV-enriched fractions, western blot analysis was performed on the fractions of increasing density for EV-enriched protein TSG101^20,21^. As shown in Fig. 1a, TSG101 was enriched in fractions 6–8 corresponding to the density of 1.10–1.16 g/mL and is consistent with the buoyant density of EVs reported in previous studies^22,23^. Hence, fractions 6, 7, and 8, that were enriched in TSG101, were pooled for further analysis. Follow-up western blot analysis confirmed the presence of the tetraspanin CD63 in both RM and CM EVs (Supplementary Fig. 1a). Western blotting of whole milk (WM) and CM EVs for high abundant milk protein casein and EV-enriched protein TSG101 confirmed the depletion of casein and enrichment of EVs in the isolated fractions (Supplementary Fig. 1b, c). Next, transmission electron microscopy (TEM) was performed on EVs isolated from both RM and CM samples. Vesicles that are characteristic of EVs in the range of 30–150 nm in diameter were observed in both the samples (Fig. 1b). Similarly, nanoparticle tracking analysis (NTA) of the samples highlighted major peaks at 145 and 135 nm for RM and CM EVs, respectively (Fig. 1c). The NTA analysis also highlighted the presence of EVs larger than 150 nm suggesting that the isolated fractions have a heterogenous mixture of EVs.

It has been postulated that milk-derived EVs may be resistant to harsh degrading conditions^24^. Hence, the stability of RM-derived EVs was examined and compared to colorectal cancer cell (LIM1215)-derived EVs (Supplementary Fig. 1d, e). From here on, EVs used in the following sections of the study have been isolated by differential centrifugation coupled with ultracentrifugation. The EVs were acidified (pH = 2) or boiled (105 °C for 15 min), then ultracentrifuged and probed for EV-enriched protein TSG101 by western blotting. Inferred by the detection of TSG101, both cancer cell- and milk-derived EVs were stable under acidic conditions (Fig. 1d). However, LIM1215 cell-derived EVs lost their stability when boiled at 105 °C. Remarkably, milk-derived EVs resisted boiling suggesting that milk-derived EVs are stable under degrading conditions and could thus potentially survive pasteurization and the harsh environment of the gastrointestinal tract. To understand the role of calcium (highly enriched in milk) in EV stability, the milk-derived EVs were treated with the calcium chelator EGTA prior to acidification and boiling (Fig. 1e). A marked decrease in the number of particles and the intensity of TSG101 was observed when EVs were treated with EGTA (Supplementary Fig. 1f–h and Fig. 1f). In agreement with this, acidification and boiling of LIM1215-derived EVs after the addition of calcium chloride led to an increase in EV stability (Supplementary Fig. 1i). Though the calcium concentration used in this study is less than the concentration in milk, addition of calcium increased stability of the EVs. An alternate explanation is that the addition of calcium may cause precipitates that also could increase the stability of EVs indirectly. Nevertheless, these observations reveal that calcium may be required for the maintenance of milk-derived EV membrane integrity under harsh conditions.

### RM and CM EVs differ in protein and RNA cargo

**Fig. 2:**
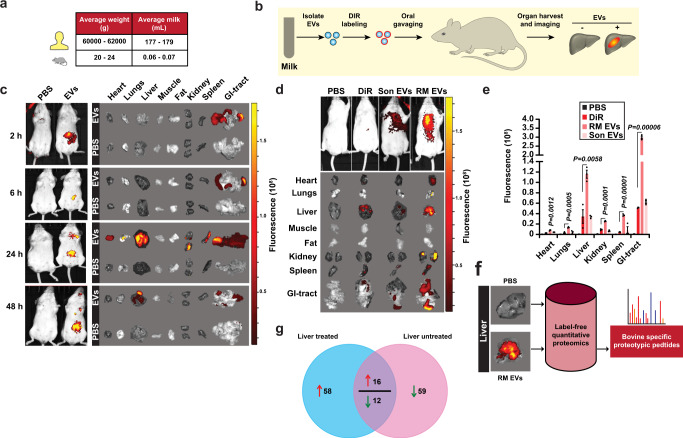
Biodistribution of orally administered bovine milk-derived EVs. **a** Schematic representation of dosage of milk-derived EVs and its physiologically relevant concentration in human and mice normalized to the body weight. **b** Schematic diagram of in vivo imaging of milk-derived EVs. **c** Female BALB/c mice were administered a single dose of 25 mg/kg DiR-labeled EVs by gavage (p.o.) and in vivo imaging of the mice after 2, 6, 24, and 48 h of EV administration were performed using IVIS Lumina XR-III (*n* = *4*). **d** Balb/c mice were orally gavaged (*p.o*.) with a single dose of DiR-labeled EVs (RM EVs) (25 mg/kg), sonicated DiR-labeled EVs (Son EVs), free DiR and PBS. After 24 h, ex vivo imaging of the tissues was performed using the In vivo imaging system (IVIS). **e** Quantification of fluorescence in mice organs (*n* = 3). **f** Schematic representation of quantitative proteomics analysis to identify bovine proteotypic peptides in mouse liver tissues. **g** Venn diagram of differentially abundant proteins in mouse liver tissue of RM EVs and PBS administered mice. The red arrow represents proteins that are of high abundance in liver tissue of RM EVs treated mice compared to PBS administered mice. The green arrow represents proteins that are of lower abundance in liver tissue of RM EVs treated mice compared to PBS administered mice. All data are represented as mean ± s.e.m. Statistical significance was determined by unpaired two-tailed *t*-test.

To determine if there were differences in EV protein content in RM and CM preparations, the purified EVs from RM and CM samples were subsequently analyzed using LC–MS/MS-based label-free quantitative proteomics. At a false discovery rate of <1%, a total of 3591 proteins were identified in both the milk-derived EV samples (Supplementary Data 1—datasets deposited in Vesiclepedia^25^) among which 1557 (43%) were detected in both EV samples. A total of 1133 and 901 proteins were exclusively detected in RM and CM EVs, respectively (Fig. 1g). In order to identify the class of proteins enriched in EVs, proteins highly abundant (>2-fold) in RM and CM EVs were subjected to functional enrichment analysis using FunRich software^26^. Although the RM and CM were equally enriched with proteins implicated in immune responses, protein transport, inflammatory responses, vesicle-mediated transport, and GTP binding, there was a marked difference between the samples in a few categories including translation, blood coagulation, innate immune responses, and structural constituents of ribosomes (Supplementary Fig. 2a–c). To examine if both the EV preparations contained EV-enriched and milk-abundant proteins, the protein list was manually inspected. As shown in Supplementary Fig. 2d, EV-enriched proteins were detected in the proteomics analysis. In addition, the proteomics analysis also revealed the presence of high abundant milk proteins (Fig. 2e) which could have resulted through the association with EV membrane (either during isolation and/or physiologically) and/or as part of the EV cargo.

Next, RNAs were isolated from EVs and enriched for small RNAs. A total of 25,684 RNAs were identified in both RM and CM EV samples (Fig. 1h, Supplementary Data 2). Among these, 2439 RNAs were significantly abundant in either of the EV samples. Analysis of small RNA, using a small RNA chip for the Bioanalyzer showed typical peaks representing the presence of various small RNA types including miRNA (425), tRNA (681), and other RNAs (Supplementary Figs. 3 and 4a). Among the RNAs that were detected in all three replicates, 8127 of them were detected in both the samples (Supplementary Fig. 4b). Significant miRNAs from both the EV samples were identified and represented (Supplementary Fig. 4c). Consistent with the proteomic analysis, the RNA cargo within the RM and CM EVs were equally enriched for immune response and metabolism (Supplementary Fig. 4d, e). However, differences in some RNA cargo implicated in cell growth and/or maintenance and transport was also observed. Despite the similarity in the core proteome and transcriptome, the differences in the cargo and the predicted functional role could be attributed to batch variations and the rigorous processing to which CM was subjected.

### Milk-derived EVs reach liver and various organs on oral administration

To determine whether milk-derived EVs can resist the harsh intestinal conditions in vivo and can be bioavailable in other organs, the biodistribution was analyzed in mice upon oral gavage. Prior to the oral administration, milk-derived EVs were labeled with the lipophilic dye DiR, subjected to OptiPrep density gradient centrifugation and the fractions were subjected to imaging. The results highlighted that dye is detectable only when bound to the EV membrane and fluoresces at the expected EV density (1.08–1.22 g/mL) (Supplementary Fig. 5a) and correlates with the presence of EV-enriched protein TSG101 (Supplementary Fig. 5b). Next, DiR-labeled EVs (25 mg/kg) were systemically administered to mice via oral gavaging (Fig. 2a, b). Following oral gavaging of DiR-labeled milk-derived EVs, whole-body in vivo imaging system (IVIS) was used to monitor the mice and image the harvested organs at different time points (2, 6, 24, and 48 h) (Fig. 2b). After 2 and 6 h, the fluorescence was only detectable in the intestine whereas at 24 h, fluorescence was also observed in liver, spleen, lungs, kidney, heart, and the gastrointestinal tract (Fig. 2c, d). After 48 h, the fluorescence signal subsided within most of the organs indicating the clearance of nanovesicles from the system. Though these results suggest that the milk-derived EV biodistribution data are consistent with a previous report^12^, we also included additional controls to examine diffusion of dye and the importance of intact EVs. As a control, an equal volume of free dye in PBS (DiR) and vehicle (PBS) controls was also orally administered. It was observed that some amount of free dye also reached the organs, but the intensity was significantly less presumably due to the absence of any lipid membrane in the samples (Fig. 2d, e). Similar to the free dye control, sonicated EVs labeled with DiR (Son EVs) exhibited less fluorescence, outlining the importance of intact EVs for absorption by the intestine. Sonication led to the significant reduction of milk-derived EVs as observed by the Western blotting for TSG101 and TEM analysis (Supplementary Fig. 6a, b). It has to be noted that upon sonication small-sized EVs were observed by TEM. Compared to the sonicated EVs, milk-derived EVs exhibited significantly higher fluorescence (Fig. 2d, e) in various organs suggesting that EVs can be absorbed by the gut and can be bioavailable in multiple organs. Next, to mimic regular daily milk intake, mice were orally administered with a daily dose of DiR-labeled RM EVs for 7 days and analyzed for biodistribution. Remarkably, all organs exhibited high intensity suggesting that milk-derived EVs are bioavailable in most of the organs (Supplementary Fig. 7a). To examine whether EVs as part of milk will have a similar effect, WM was spiked with labeled EVs and orally administered to examine the biodistribution. In concordance with the previous observations, the fluorescence could be detected in liver, kidney, spleen, and GI-tract (Supplementary Fig. 7b, c). Overall, these results suggest that the lipid membrane of bovine milk-derived EVs is important for surviving passage through the gut and localize to various organs.

Whilst the results obtained from the biodistribution experiments were interesting, the observations require cautious interpretation as they are dependent on the lipophilic dye DiR. To exclude the possibility that the fluorescence observed in the organs could have resulted from EV membrane fragments and to validate the bioavailability, a label-free quantitative proteomics analysis was performed in order to examine whether proteins of bovine origin could be detected in the liver tissue of mice orally administered with milk-derived EVs (Fig. 2f). Mice were administered daily with RM EVs (25 mg/kg) or PBS for a duration of 7 days. On the final day, the liver was collected. Liver tissue lysates were subjected to proteomics analysis and the resulting MS/MS spectra were searched against mouse and bovine RefSeq protein database. Peptides exclusive to liver tissue from RM EV-treated mice (at least in 2 replicates) were subjected to BLAST, and bovine-specific proteotypic peptides^27^ (exclusive to Bovidae genome) were identified and analyzed. A total of 129 proteotypic peptides (Supplementary Data 3) corresponding to 110 bovine proteins were detected in mouse liver tissues. These results suggest that milk-derived EVs can protect cargo against the harsh environment of the GI-tract and deliver the encapsulated contents to various organs. Furthermore, oral administration of bovine milk-derived EVs modified the proteome of the liver tissue (Fig. 2g and Supplementary Data 4). Upon ingestion of milk-derived EVs, a total of 145 mouse proteins were differentially abundant (at least 2 replicates) in mouse liver tissue suggesting that oral administration of bovine milk-derived EVs regulated the proteome of the host liver marginally. Taken together, these results suggest that diet-based milk-derived EVs can survive the gut, reach multiple organs and regulate the protein abundance in the host tissue.

### Milk-derived EVs reduce tumor burden and attenuate cancer-induced weight loss

**Fig. 3:**
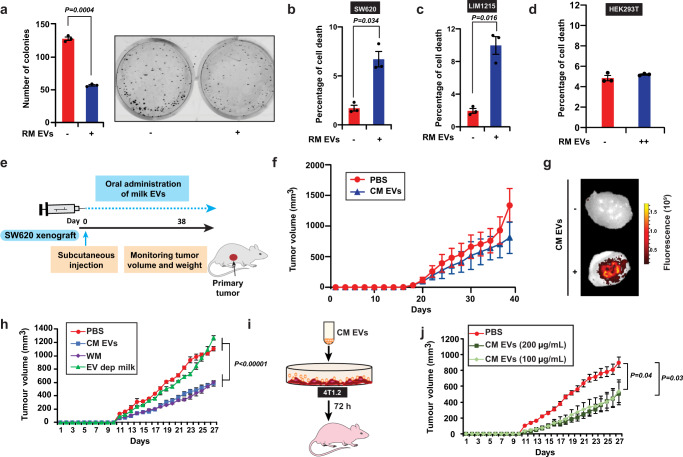
Milk-derived EVs reduce tumor burden. **a** SW620 colorectal cancer cells were treated with RM EVs (20 μg/mL) and colonies were quantified (*n* = 3). **b** Apoptosis of SW620 colorectal cancer cells at 72 h after treatment with RM EVs (20 μg/mL; *n* = 3). **c** Apoptosis of LIM1215 colorectal cancer cells at 72 h after treatment with RM EVs (20 μg/mL; *n* = 3). **d** Apoptosis of human embryonic kidney (HEK293) cells at 72 h after treatment with RM EVs (++ = 50 μg/mL; *n* = 3). **e** Schematic representation of colorectal cancer xenograft experiment. **f** Tumor volume of SW620 xenografts measured after oral administration of PBS and CM EVs (25 mg/kg; *n* = 3). **g** SW620 tumor-bearing mice were orally administered with a single dose of DiR-labeled EVs (25 mg/kg). IVIS imaging of the harvested tumor tissue after 24 h of EVs administration is displayed. **h** Tumor volume of 4T1.2 bearing mice after oral administration of PBS, CM EVs (25 mg/kg), WM (70 µL), and EV-depleted milk (*n* = 5). **i** 4T1.2 cells were treated with CM EVs (100 and 200 µg/mL) for 72 h and injected into Balb/c mice. **j** Tumor volume of 4T1.2 cells treated with and without CM EVs (*n* = 5). All data are represented as mean ± s.e.m. Statistical significance was determined by unpaired two-tailed *t*-test.

Though the biodistribution of milk-derived EVs is interesting, additional evidence on the functional effects of these orally ingested EVs would establish the dietary EV relevance in cross-kingdom communication and mammalian physiology. Hence, the functional effects of milk-derived EVs were examined on cancer cells using clonogenic and cell death assays. Treatment of SW620 colorectal cancer cells with milk-derived EVs (20 µg/mL) resulted in a significant decrease in the number of colonies in the treatment group (Fig. 3a). Similarly, cell cycle analysis confirmed the induction of cell death in colorectal cancer cells (LIM1215 and SW620) when treated with milk-derived EVs (Fig. 3b, c). Interestingly, no significant difference in cell death was observed when immortalized embryonic kidney (HEK-293T) cells were treated with milk-derived EVs (Fig. 3d). Hence, it can be speculated that the sensitivity of milk-derived EVs may vary based on the cell type. Nevertheless, these results suggest that milk-derived EVs have anti-proliferative effects on cancer cells in vitro as shown with previous studies^13^.

To determine if milk-derived EVs could affect tumor growth in vivo, nude mice were injected with SW620 cells (2.5 × 10^6^) subcutaneously (s.c.). Simultaneously, CM EVs were administered by oral gavaging (25 mg/kg, Fig. 3e). Upon daily oral administration of milk-derived EVs, tumor growth was reduced in the treatment groups (Fig. 3f). To examine whether the orally administered bovine milk-derived EVs reach the tumor, mice bearing subcutaneous tumors were orally gavaged with PBS or DiR-labeled bovine milk-derived EVs and the tumor tissue was harvested and subjected to IVIS imaging. As shown in Fig. 3g, bovine milk-derived EVs indeed reach the tumor after 24 h of oral delivery. Consistent with in vitro observations, these results suggest that milk-derived EVs possess anti-tumor activity.

Next, the effect that WM and CM EVs administration had on the bodyweight condition was assessed. The physiological mice concentration of average human milk consumption (~179 mL) is estimated as 70 µL but several individuals consume more than 500 mL daily, especially children^28^. Infact, several countries recommend adults to consume more than 700 mL of milk daily. In addition, these concentrations have not taken into account the dairy products including cheese, butter, yogurt, and ice cream that may contains milk-derived EVs. Hence, the precise physiological concentration of milk and dairy product consumption and normalization with body weight depends on the specific individual (adults and children). Nevertheless, oral gavage of WM (double the physiological dose for 179 mL to 140 µL) or CM EVs did not alter the weight of the mice (Supplementary Fig. 8a). This observation is consistent with previous reports^12,29^ where no bodyweight loss or damage to tissues or changes in plasma cytokine profile was observed. In addition to toxicity, it is important to examine whether WM also has anti-tumor activity and what proportion of this functional activity is contributed by the EV fraction. To address this and to check the reproducibility of the anti-tumor activity, 4T1.2 breast cancer cells were implanted in Balb/c mice and orally administered with PBS control, WM (70 µL), CM EVs, and the EV depleted milk. Interestingly, WM and CM EVs were able to reduce the primary tumor burden significantly (Fig. 3h). However, the EV-depleted milk did not significantly reduce the primary tumor burden suggesting that the EV fraction is solely responsible for the anti-tumor activity. Next, to examine whether milk-derived EV treatment of cancer cells, in vitro, would influence primary tumor growth, 4T1.2 cells were treated for 72 h with CM EVs (100 and 200 µg/mL) and injected in Balb/c mice (Fig. 3i). Remarkably, one dose of CM EV treatment was sufficient to significantly reduce tumor growth (Fig. 3j).

The presence of metastasis and cancer cachexia significantly reduces patient survival and contributes to the majority cancer-related deaths^30^. Whilst milk-derived EVs can reduce the primary tumor burden, it is unclear whether they influence these two factors. Hence, the role of orally administered milk-derived EVs in the progression of cancer-associated weight loss was examined using C-26 colorectal tumor models. Immunocompetent CD2F1 mice were pre-treated for 4 days by orally gavaging RM and CM EVs (25 mg/kg). On day 5, the mice were s.c. inoculated with C-26 tumor cells along with continual daily treatment with milk-derived EVs (Supplementary Fig. 8b). A rapid loss in weight was observed in the control group after day 18 whereas the CM EVs administered group exhibited stable weights (Supplementary Fig. 8c). Surprisingly, unlike CM EVs, RM EVs did not significantly prevent tumor-associated weight loss. The preservation of weight in the pair-fed (PF) control group showed that the loss of body mass in the control C-26 tumor mice was not due to variance in food intake. Importantly, the survival of the mice significantly increased when orally administered with RM or CM EVs (Supplementary Fig. 8d). Reasons for varying effects of CM and RM EVs in loss of body mass and the beneficial effect of RM EVs in survival despite no significant change in body mass is unclear and needs to be examined in follow-up studies.

### Milk-derived EVs augment pancreatic cancer liver metastasis

**Fig. 4:**
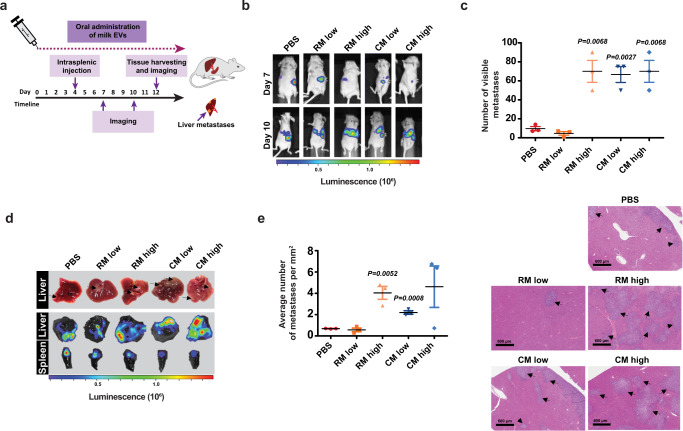
Bovine milk-derived EVs increase metastasis of pancreatic cancer cells. **a** Schematic representation of intrasplenic injection of KPC pancreatic cancer cells and treatment timeline with milk-derived EVs. **b** Representative whole-body imaging of luciferase signal on day 7 and 10 following intrasplenic injection of luciferase-KPC cells and during daily treatment with RM and CM EVs (low dosage: 25 mg/kg; higher dosage: 50 mg/kg). **c** Number of visible metastases in control and milk-derived EVs fed mice (*n* = 3). **d** Pathological and luminescence-based examination of liver metastases and quantification of visible metastases on the surface of the tissue (*n* = 3). Black arrows point at KPC metastases visible at the surface of the organ. **e** Quantification of liver metastases normalized to liver surface area in H&E sections. Black arrows point at metastases in the liver tissue. All data are represented as mean ± s.e.m. Statistical significance was determined by unpaired two-tailed *t*-test.

Metastasis is a major cause of cancer morbidity and mortality. Since milk-derived EVs reduced the primary tumor burden, we hypothesized that milk-derived EVs can attenuate cancer metastasis. To study the effect bovine milk-derived EVs have on metastasis, mice were subjected to intrasplenic injections with (KPC) pancreatic cancer cells, expressing luciferase (Fig. 4a), to effectively mimic the early stages of metastatic spread as described previously^31^. To examine whether higher dosages are curative, the mice were administered daily with a low (25 mg/kg) and high dose (50 mg/kg) of milk-derived EVs. Whole-body imaging of luciferase by IVIS was also performed on day 3 and 6 post-intrasplenic injections (Fig. 4a, b) to monitor metastatic spread. On day 10, mice were sacrificed, and metastatic burden was assessed via IVIS imaging and quantification of visible metastases at the surface of organs. Intriguingly, oral administration of bovine milk-derived EVs increased liver metastasis, being one of the most notable sites for metastatic dissemination in pancreatic cancer, in a dose-dependent manner (Fig. 4c). Contrary to our hypothesis, luciferase imaging of liver and quantification of visible metastases on the surface of the tissue suggested that milk-derived EVs induce liver metastasis (Fig. 4d). Furthermore, quantification of metastases on hematoxylin and eosin (H&E) liver sections (Fig. 4e) confirmed that mice administered with high dosage of milk-derived EVs exhibited a marked increase in metastatic spread compared to the control group. Overall, for the first time, these results suggest that oral delivery of milk-derived EVs in mice, bearing pancreatic cancer, can promote metastatic colonization of pancreatic cancer cells to the liver.

### Milk-derived EVs accelerate breast cancer lung metastasis

**Fig. 5:**
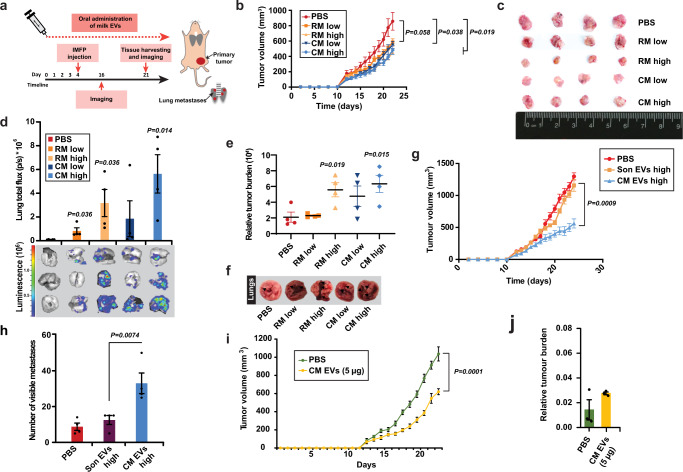
Bovine milk-derived EVs increase metastasis of breast cancer cells. **a** Schematic representation of intramammary fat pad injections (IMFP) of 4T1.2 breast cancer cells and treatment timeline with milk-derived EVs. **b** Primary breast tumor volume in immunocompetent mice treated with milk-derived EVs (low dosage: 25 mg/kg; higher dosage: 50 mg/kg; *n* = 4). **c** Harvested primary tumor size from mice treated with PBS and milk-derived EVs is shown. **d** Quantification of ex vivo imaging of lungs from metastatic breast cancer models treated with PBS and milk-derived EVs. Total lung flux to quantify breast cancer metastasis to lungs is depicted (*n* = 4). **e** Relative metastatic tumor burden is depicted (*n* = 4). **f** Pathological examination of lung metastases on the surface of the tissue. **g** Primary breast tumor volume in immunocompetent mice orally administered with PBS, sonicated and intact milk-derived CM EVs (higher dosage: 50 mg/kg; *n* = 5). **h** Pathological examination of lung metastases and quantification of visible metastases on the surface of the lungs from mice orally administered with PBS, sonicated and intact milk-derived CM EVs (*n* = 5). **i** Primary breast tumor volume in immunocompetent mice orally administered with PBS (*n* = 8) and CM EVs (5 µg; *n* = 9). **j** Relative metastatic tumor burden is depicted (*n* = 3). Mice exclusion reason: for measuring the relative tumor burden, 3 mice lungs were used randomly; the remaining mice lungs were used to isolate mCherry positive cells for a different experiment. All data are represented as mean ± s.e.m. Statistical significance was determined by unpaired two-tailed *t*-test.

As intriguing metastasis results were obtained from pancreatic cancer models in immunocompromised mice, we sought to assess whether similar results could be obtained in a different metastatic setting in immunocompetent models. Hence, milk-derived EVs were administered to immunocompetent murine syngeneic breast cancer models. Balb/c mice were injected with murine breast cancer cells 4T1.2 cells to the intramammary fat pad and were orally administered with milk-derived EVs daily (Fig. 5a). Consistent with previous results in CRC and breast cancer cells, milk-derived EVs significantly reduced the primary tumor burden (Fig. 5b, c). Irrespective of the reduction in primary tumor growth, breast cancer metastasis to the lung was significantly increased in mice administered with bovine milk-derived EVs (Fig. 5d). Though the body weight of the mice remained unaffected, the relative metastatic tumor burden (Fig. 5e) and macroscopic lung metastasis was increased in mice administered with both the milk-derived EVs (Fig. 5f). It must be noted that metastatic assays were not normalized to the primary tumor burden. To examine whether the immune cells have a functional role in primary tumor reduction and accelerated metastasis, FACS analysis was performed. However, no active role could be attributed to immune cells, as FACS analysis of circulating or infiltrating immune cells in the tumor microenvironment revealed no significant changes between the control and treatment groups (Supplementary Figs. 9, 10, and 11).

To understand the importance of intact EVs in cross-species communication, tumor burden and the metastasis, an additional experiment was performed with oral administration of intact and sonicated EVs. Whilst oral administration of intact CM EVs reduced the primary tumor burden significantly, sonicated CM EVs were unable to reduce the primary tumor similar to untreated controls (Fig. 5g). Consistent with these findings, oral administration of sonicated EVs did not accelerate metastasis while intact EVs significantly increased the metastatic burden (Fig. 5h and Supplementary Fig. 12a). Next, we examined whether low levels of CM EVs can also have a functional effect. Interestingly, low levels of CM EVs (5 µg) were able to reduce the primary tumor burden significantly (Fig. 5i and Supplementary 12b). However, low levels of CM EVs could not accelerate metastasis significantly (Fig. 5j). These results suggest that the primary tumor burden reduction is not dependent on the concentration of CM EVs while the pro-metastatic effect is. To our knowledge, this is the first report illustrating that reduction in primary tumor burden can also accelerate metastasis and emphasizes caution on the interpretation of results from xenograft models.

### Milk-derived EVs induce cellular senescence and epithelial-to-mesenchymal transition

**Fig. 6:**
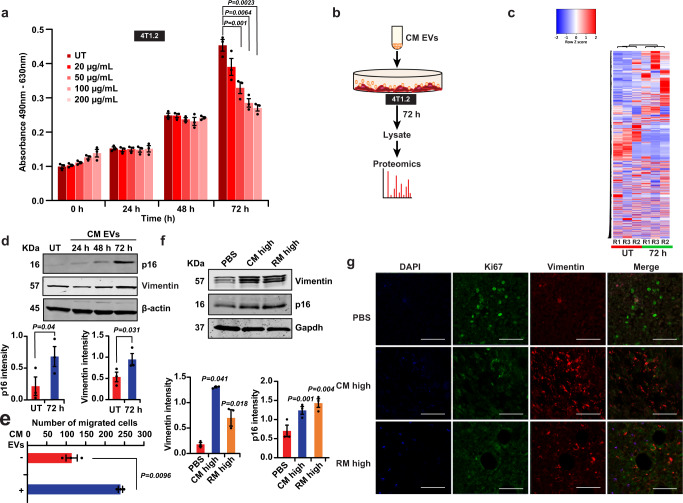
Milk-derived EVs induce cellular senescence and epithelial-to-mesenchymal transition in cancer cells. **a** MTS-based proliferation assay on 4T1.2 breast cancer cells with increasing concentration of milk-derived EVs shows a dose-dependent reduction in metabolic activity at 72 h (dosage = 20, 50, 100 and 200 μg/mL; *n* = 3). **b** Schematic representation of proteomics analysis performed on 4T1.2 breast cancer cells incubated with 100 μg/mL of CM EVs for 72 h. **c** Heatmap highlighting the differential abundance of proteins in 4T1.2 breast cancer cells upon treatment with milk-derived EVs. **d** Western blot analysis shows the increase in the senescence marker p16 and cytoskeletal protein Vimentin upon treatment of 4T1.2 cells with milk-derived EVs in vitro *(n* = *3)*. **e**, Treatment of 4T1.2 cells with milk-derived EVs increases the migration of breast cancer cells (*n* = *3*). **f** Western blotting of primary tumor isolated from mice orally administered with or without milk-derived EVs confirmed the upregulation of senescence regulator p16 and cytoskeletal protein Vimentin in vivo *(n* = 3*)*. **g** Immunofluorescence of primary tumor sections shows upregulation of Vimentin and reduction in cell proliferation marker Ki67 in mice orally gavaged with milk-derived EVs *(n* = 3). Scale bar represents 100 µM. All data are represented as mean ± s.e.m. Statistical significance was determined by two-tailed *t*-test.

To understand how milk-derived EVs regulate this opposing role of reducing primary tumor burden and accelerating metastasis, a series of experiments were conducted. First, the effect of milk-derived EVs on cell viability was examined. As shown in Supplementary Fig. 13, treatment of cancer cells with pan-caspase inhibitor QVD or necroptosis inhibitor necrostatin did not increase the cell viability after milk-derived EV treatment. These data suggest that milk-derived EVs reduce the proliferation of cancer cells independent of caspase-dependent apoptosis and necroptosis. To examine the role of autophagy-induced cell death, CRISPR-Cas9 knockout of the autophagy regulator ATG5 was established (Supplementary Fig. 14a). Treatment of ATG5^−/−^ cells with milk-derived EVs or Cas9 cells with the autophagy inhibitor Bafilomycin A did not increase the cell viability of cancer cells (Supplementary Fig. 14b). Hence, these data suggest that the anti-proliferative effect of milk-derived EVs is not dependent on autophagy. From these results, it was evident that milk-derived EVs do not orchestrate anti-tumor effects via apoptosis. Next, an MTS-based proliferation assay was performed with cancer cells with increasing concentration of milk-derived EVs. As shown in Fig. 6a, a dose-dependent reduction in metabolic activity was observed at 72 h. However, no significant difference in metabolic activity could be observed between 100 and 200 μg/mL of milk-derived EVs.

To understand how milk-derived EVs reduce the proliferation of breast cancer cells, a proteomic analysis was performed on 4T1.2 cells incubated with milk-derived EVs for 72 h (Fig. 6b). Heatmap of the proteomics analysis highlighted the differential abundance of proteins upon treatment with milk-derived EVs (Fig. 6c). More specifically, 1118 proteins were differentially abundant when 4T1.2 cells were treated with milk-derived EVs (Supplementary Data 5). A total of 442 and 676 proteins were of high and low abundance, respectively, upon incubation of 4T1.2 cells with milk-derived EVs. Next, using FunRich^32^ analysis, the proteomics results were analyzed to identify protein classes that are enriched in biological processes, molecular function, and biological pathways. The analysis highlighted that incubation of milk-derived EVs depleted the abundance of proteins implicated in translation and mRNA processing (Supplementary Fig. 15a). On the contrary, proteins implicated in epithelial cell migration were enriched (though not significant) in 4T1.2 cells incubated with milk-derived EVs. The analysis on molecular functions highlighted that proteins involved in cadherin binding, GTPase activity and RNA binding were decreased upon milk-derived EV treatment. Whereas, the proteins implicated in ATP and calmodulin binding were enriched when 4T1.2 cells were incubated with milk-derived EVs (Supplementary Fig. 15b). Consistent with the MTS proliferation assay and the functional enrichment analysis, treatment of 4T1.2 cells with milk-derived EVs reduced the abundance of proteins implicated in G2/M checkpoints, mRNA splicing and separation of sister chromatids.

As the proteomic analysis highlighted alterations in RNA processing, protein translation and cell cycle progression, the role of cellular senescence was examined. Consistent with the MTS proliferation assay, treatment of 4T1.2 cells with milk-derived EVs induced cellular senescence as indicated by the upregulation of the senescence regulator p16 (Fig. 6d). In agreement with Western blot results, total number of β-galactosidase positive cells was significantly increased when milk-derived EVs were incubated with 4T1.2 cancer cells (Supplementary Fig. 16a). Next, to examine whether the induction of senescence is due to DNA damage, comet assay was performed. However, 4T1.2 cancer cells incubated with milk-derived EVs did not exhibit DNA damage, suggesting that the EV cargo and/or associated molecules induced senescence in the cancer cells, in a DNA-damage-independent manner (Supplementary Fig. 16b). Nevertheless, these results suggest that milk-derived EVs induce cellular senescence in cancer cells and reduce the primary tumor burden. However, it is unclear how the cancer cells become pro-metastatic in spite of induction of senescence. It has been previously proposed that cancer cell-derived EVs can drive a pre-metastatic niche^33^. Similarly, studies have also highlighted that senescent cells secrete more proteases like MMPs and EVs that can increase the invasion of cancer cells^34–37^. Hence, we examined whether MMPs and EVs in the senescent cells have a role in accelerating metastasis. Manual analysis of the proteomics data of 4T1.2 cells treated with or without CM EVs for MMPs highlighted no changes in the abundance of MMP1, 3, 9, 10, and 14 (Supplementary Fig. 16c). However, one of critical proteases MMP2^34^ was not detected and hence western blotting for the protease MMP2 in primary tumor lysates was performed. The analysis revealed no significant changes in the levels of MMP2 upon induction of senescence (Supplementary Fig. 16d). Next, the role of senescent cell-secreted EVs in accelerating migration was examined. CM EVs were incubated with 4T1.2 breast cancer cells for 72 h to induce senescence. After 24 h, the EVs were collected from the senescent cancer cells (Supplementary Fig. 16e). EVs secreted by senescent cells were able to increase the migration of the less invasive MCF7 breast cancer cells marginally (Supplementary Fig. 16f). Though these results can speculate that senescent cell-derived EVs can potentially induce pro-metastasis, we next examined the cell-intrinsic alterations that can increase metastasis.

Epithelial-to-mesenchymal transition (EMT) has long been associated with metastasis and hence, we examined whether milk-derived EVs induce EMT in cancer cells. Western blot analysis confirmed the increase in the mesenchymal marker and cytoskeletal protein Vimentin, in 4T1.2 cells, upon treatment with milk-derived EVs (Fig. 6d). Consistent with the upregulation of Vimentin, western blot analysis confirmed the upregulation of EMT regulators Twist and Snail (Supplementary Fig. 17a) when 4T1.2 cells were treated with milk-derived EVs. Consistent with the proteomic analysis, total Stat3 was downregulated in 4T1.2 cells treated with milk-derived EVs. No significant difference could be observed for autophagy regulator p62, Mapk and p53. To examine whether induction of EMT results in enhanced migration, 4T1.2 cells were treated with milk-derived EVs and subjected to trans-well migration assay. As shown in Fig. 6e, milk-derived EVs induced migration in the 4T1.2 cells despite induction of cellular senescence. Similarly, milk-derived EVs were able to increase the wound healing ability of the less invasive MCF7 breast cancer cells (Supplementary Fig. 17b). Hence, these results suggest that milk-derived EVs induce EMT in cancer cells and increase their invasiveness.

While the mechanistic insights for the opposing effects of milk-derived EVs in reducing primary tumor burden and accelerating metastasis can be explained by cellular senescence and EMT, the in vivo relevance of these observations need to be examined. Hence, to verify the induction of senescence and EMT in vivo, the primary tumor was subjected to western blotting and immunofluorescence. In accordance with the in vitro observations, Western blotting of primary tumor, isolated from mice orally administered with or without milk-derived EVs, confirmed the upregulation of senescence regulator p16 and cytoskeletal protein Vimentin (Fig. 6f). Immunofluorescence of primary tumor sections also confirmed the upregulation of Vimentin and reduction in cell proliferation marker Ki67 in mice orally gavaged with milk-derived EVs (Fig. 6g). Taken together, these data suggest that milk-derived EVs induce senescence and EMT in the primary tumor that accounts for the reduction in the tumor size and accelerated metastasis, respectively.

### Surgical resection of primary tumor reverses the pro-metastatic effects of milk-derived EVs

**Fig. 7:**
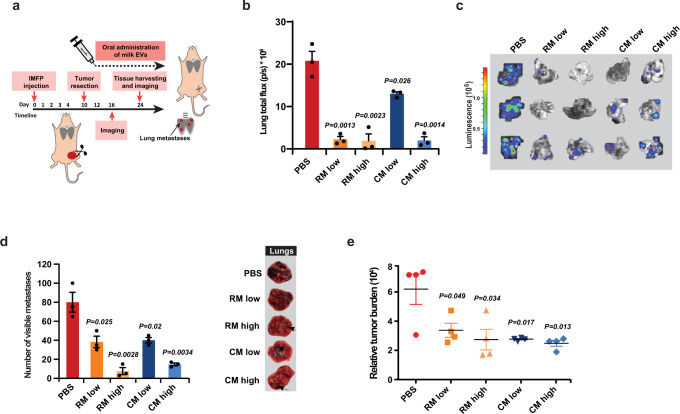
Resection of primary tumor significantly reduces metastasis. **a** Schematic representation of tumor resection and treatment timeline with milk-derived EVs. **b** Total lung flux to quantify breast cancer metastasis is depicted (*n* = 4). **c** Lungs were excised from the mice at the endpoint and subjected to bioluminescence. **d** Pathological examination of lung metastases and quantification of visible metastases on the surface of the tissue (*n* = 4). **e** Relative metastatic tumor burden is depicted (*n* = 4). All data are represented as mean ± s.e.m. Statistical significance was determined by unpaired two-tailed *t*-test.

From the mechanistic insights, it is evident that the primary tumor is critical for the accelerated metastatic effect induced by milk-derived EVs. Hence, it was hypothesized that in the absence of primary tumor, milk-derived EVs can induce senescence in the cancer cells that have already seeded in the secondary organs. To further understand the role of the primary tumor in milk-derived EV-induced metastasis, 4T1.2 breast cancer cells were injected to the intramammary fat pad of immunocompetent mice and the primary tumor was resected after 10 days (Fig. 7a). Two days after surgery, the mice were orally administered with milk-derived EVs. Interestingly, total flux (Fig. 7b) and luminescence (Fig. 7c) of lungs harvested from control and EV administered groups show a reversal in the trend of the metastatic burden. Compared to the PBS control, all groups administered with milk-derived EVs had significantly reduced metastasis. The results were further corroborated by quantification of the number of visible metastases on the surface of the lungs (Fig. 7d) and relative metastatic tumor burden (Fig. 7e). Hence, it can be concluded that after surgery, oral administration of milk-derived EVs attenuates the metastatic effect of highly aggressive breast cancer cells, and the context must be taken into consideration. These results suggest that milk-derived EVs can be beneficial to metastatic cancer patients who have undergone surgery to remove the primary tumor.

## Discussion

Our results demonstrate that bovine milk-derived EVs can be absorbed, bioavailable in various organs, and can regulate the proteome profile of liver tissue and thus supporting the concept of cross-species communication. EVs provide an efficient means to protect the otherwise fragile cargo against harsh degrading conditions of the intestinal tract and thus enhancing the possibility of delivery of the bioactive contents to the host following oral ingestion^17,38,39^. While the striking findings in this study, provide some insights on the role of dietary EVs in cross-species communication and in cancer progression, we have not used any non-milk EV controls. This is mainly because our study predominantly focused on cross-species communication with milk-derived EVs. Milk is one of the highly consumed beverages by humans that contains high amounts of EVs. However, it must be noted that very few dietary sources may have as high concentrations of EVs as milk. Hence, though cross-species communication is plausible for milk-derived EVs, we emphasize caution to not relate this finding to every dietary source most of which normally contains a low concentration of EVs. For instance, beer is one of the highly consumed beverages in the world. Orally administered DiR-labeled EVs isolated from six brands of filtered beer (pooled—physiological dosage) was not bioavailable in the mice due to low concentrations (Supplementary Fig. 18a). Interestingly, orally administered high doses of DiR-labeled yeast EVs (25 mg/kg) were bioavailable in multiple organs (Supplementary Fig. 18b). These results argue for the fact that any EVs at high concentrations can survive the gut and be bioavailable. However, the concentration of yeast EVs used in this study is not physiologically relevant and it is estimated that more than 7000 filtered beers need to be consumed to reach this concentration.

It has been proposed that EVs could survive the gut and can circulate in blood either through transendocytosis or diffusion through epithelial barrier^12,40^. In support of this speculation, LPS positive bacterial EVs have been detected in circulation in patients with intestinal barrier dysfunction^40^. However, no experimental evidence, in in vivo conditions, exists to confirm that EVs can survive the gut and reach circulation through transendocytosis and/or diffusion. It is unclear as whether what fraction of the EVs are damaged or lost through epithelial cell uptake or disposed as waste and what proportion can survive the gut. Clearly, additional research is needed to unravel the mechanism of by which EVs can survive the gut and be bioavailable in the consuming organism.

**Fig. 8:**
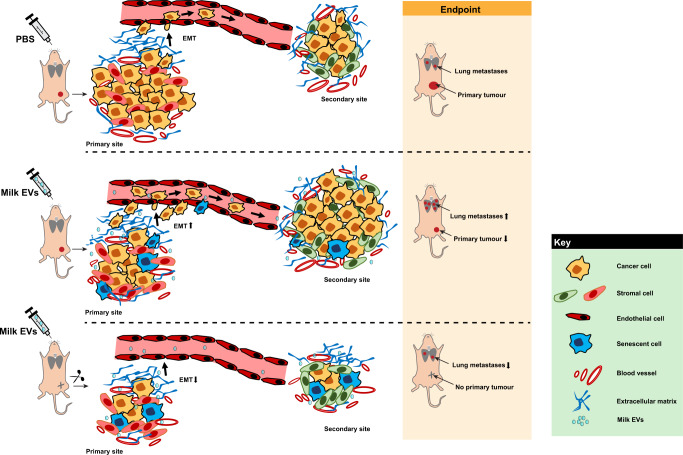
Summary of the study. Orally administered milk-derived EVs can reduce the primary tumor burden and accelerate metastasis in a context-dependent manner. Milk EVs can induce senescence and EMT in the primary tumor thereby allowing for reduction in primary tumor and acceleration of metastasis. When the primary tumor is resected, milk-derived EVs induce senescence in the metastatic site thereby reducing the proliferation of cells seeded in distant sites.

Though preliminary analysis from this study suggest that milk-derived EVs are stable under harsh degrading conditions, additional work is needed to characterize this in vivo. For instance, the stability of RNA, other luminal and membrane proteins in the milk-derived EVs need to be studied. Furthermore, the uptake and functionality of the EVs after incubated with harsh degrading conditions need to be examined. As EVs are exploited as drug delivery vehicles^41^, the stability and bioavailability of EVs are critical factors. Recently, milk-derived EVs packaged with chemotherapeutic drugs have been shown to reduce the primary tumor burden^13^. Though our data with milk-derived EVs alone had a significant inhibitory effect on primary tumor burden, to our surprise, we identified an interesting role of these membranous vesicles in inducing metastasis. Further to this, we also investigated the impact of these vesicles on lung metastasis in breast cancer models after resection of the primary tumor. Interestingly, we observed a significant decrease in the extent of metastasis in a dose-dependent manner when the milk-derived EVs were orally delivered after surgically removing the primary tumor (Fig. 8). Our data demonstrate the context-dependent effects of milk-derived EVs and the potential benefit of cross-species EVs in cancer treatment. For instance, milk-derived EVs can be utilized to treat metastatic cancer patients who have undergone surgery to remove primary tumor. Although additional research is needed to explore the potential implications of milk-derived EVs in cancer therapy, the lack of successful metastatic therapeutic interventions argues for its consideration.

This is the first report to our knowledge that shows reduced tumor burden but accelerated metastasis thereby raising alarm over the use of xenograft models for cancer studies. However, one of the outstanding questions in this study relates to the identification of the precise protein/RNA cargo in the EVs that is instrumental in the opposing effect of reduced tumor burden and accelerated metastasis. The identification of one or few cargo molecules in EVs to a specific phenotype is a hard task, especially for bovine milk-derived EVs. Normally knockdown or knockout of the gene of interest/EV cargo (e.g., delta catenin) in the host cell will be performed, the EVs will be isolated from the delta catenin positive and negative cells and the exact function can be examined. If the EVs elicit different phenotypes on the recipient cells, it can be suggested that the functional effect can be dependent on delta catenin in EVs. However, similar experiments are extremely difficult in bovine milk-derived EVs as transgenic cows are required. An alternate strategy is the use of inhibitors or knockout genes in the recipient cells. A significant concern in this approach in the use of inhibitors or knockout of genes is that the perturbations merely alter the recipient cells uptake efficiency of EVs and have little to do with the precise molecule(s) in question. Regardless, additional research is needed to identify the key molecules that drive these opposing effects in the cancer models. Proteomic analysis of the milk-derived EVs detected proteins that are present in high abundance in milk (Supplementary Fig. 2e). Hence, it could be argued that the functional effect pertaining to cancer progression may be mediated by the high abundant milk proteins, at least in part. Whilst WM by itself had a similar effect as EVs, EV depleted milk that is enriched in many high abundant proteins did not have a functional effect (Fig. 3h). This result suggests that intact EVs either by carrying the luminal cargo or through transferring associated molecules could be the driving factor for the observed phenotype in this study.

From various results obtained in this study, it can be suggested that the primary tumor reduction can mainly be attributed to the induction of senescence. Remarkably, one dose of milk-derived EV treatment in vitro (Fig. 3i, j) was sufficient to reduce the primary tumor burden in vivo. However, the augmented metastasis could be attributed to multiple effects such as EMT and senescence. As shown in Supplementary Fig. 16f, senescent cell can secrete EVs which can increase the migration of the cancer cells. Hence, it is possible that these senescent cells could secrete more pro-metastatic EVs, as reported previously^36,42^, and accelerate pre-metastatic niche. This possibility has not been tested in vivo in this study and hence need to be examined in follow-up projects.

Nevertheless, we were able to demonstrate that milk-derived EVs are bioavailable in consuming host organisms and could exert significant functional effects even at very low concentrations (5 µg). Whilst it is encouraging that milk-derived EVs have tumor growth-suppressive effects (at least in part), we emphasize caution as the dosage and time of treatment may have profound influence on the outcome. Encouragingly, low concentrations of milk-derived EVs (5 µg) were sufficient to reduce the primary tumor burden but were unable to accelerate metastasis. Milk-derived EVs at 25 mg/kg have been used previously to deliver chemotherapeutic drugs to treat pre-clinical models^12^, however, at that concentration our data suggests that milk-derived EVs can accelerate metastasis. Based on these observations, it can be suggested that milk-derived EVs have anti-cancer properties if the dosage is controlled and has the potential to be exploited in therapeutic applications in a context-dependent manner. In this work, we report an important finding that oral consumption of milk-derived EVs decreased the primary tumor growth but paradoxically increased lung and liver metastasis. It can be speculated that our findings have direct clinical implications as high consumption of milk may increase the metastatic burden in cancer patients with intact primary tumors. On the contrary, it can be speculated that milk consumption may be beneficial to breast cancer patients when the primary tumor is surgically removed. However, follow-up pre-clinical and epidemiological studies are needed to test these hypotheses. Clearly, additional research is needed to harness the cancer suppressive benefits of milk-derived EVs by itself and in combination with other drugs.

## Methods

### Cell culture

4T1.2, LIM1215 and MCF7 cells were cultured in 150 cm^2^ tissue culture flasks (BD Falcon™) in MEM alpha, RPMI, and DMEM (GIBCO, Life Technologies) medium, respectively. The culture medium was supplemented with 10% (v/v) FCS (GIBCO, Life Technologies) and 100 U/mL of penicillin–streptomycin (GIBCO, Life Technologies). The cells were incubated at 37 °C with 5% CO_2_.

### Isolation of EVs by differential centrifugation and ultracentrifugation

RM from pasture-fed Holstein cows during the mid-lactation period was obtained from DEPI Ellinbank. Commercial cow milk (CM) was obtained from local grocery store by pooling five different brands together. La Trobe Animal Ethics committee approved all experimental protocol used in this study. Milk samples were centrifuged immediately (within 3 h of procurement) at 2000 × *g* for 15 min at 4 °C. The samples were stored in −80 °C for further processing. The defatted samples were then subjected to successive centrifugations as described previously^11^. The pellet obtained was resuspended in PBS and a final washing step was performed by ultracentrifugation at 100,000 × *g* for 1 h at 4 °C (Beckman Coulter: TLA-55 rotor). The obtained EV pellet was resuspended in 0.5 mL PBS and stored at −80 °C.

For 4T1.2 cells-derived EV isolation, cells were first allowed to reach 70–80% confluence. The cells were then washed with PBS and fresh media containing EV depleted FCS was added. After 24 h of incubation, the conditioned media was subjected to differential centrifugation (500 × *g* for 10 min, 2000 × *g* for 20 min, and 10,000 × *g* for 30 min). The obtained supernatant was further spun at 100,000 × *g* for 1 h at 4 °C. The pellet was the washed with PBS before subjecting to ultracentrifugation at 100,000 × *g* for 1 h at 4 °C. The obtained pellet was resuspended in PBS and stored in −80 °C.

### Density gradient ultracentrifugation

Density gradient ultracentrifugation was performed as described previously with modifications^43^. A discontinuous iodixanol gradient consisting of 40% (w/v), 20% (w/v), 10% (w/v), and 5% (w/v) solutions of iodixanol was prepared by diluting a stock solution of OptiPrep (60% (w/v) aqueous iodixanol from Sigma life Sciences) in 0.25 M sucrose/10 mM Tris, pH 7.5. The fractions were layered with milk-derived EVs isolated by differential centrifugation coupled with ultracentrifugation, resuspended in the OptiPrep solution, and overlaid onto the top layer. A control tube consisting 3 mL of each 40%, 20%, 10% and 5% solutions were also prepared. The tubes were subjected to ultracentrifugation at 100,000 × *g* for 18 h at 4 °C (Beckman Coulter: SW-28 rotor). Pellets were then washed with 1 mL of PBS and the supernatant was removed with 2 successive ultracentrifugation at 100,000 *g* for 1 h at 4 °C (Beckman Coulter: TLA-55 rotor) and resuspended in 200 µL before being stored at −80 °C.

### Western blotting and antibodies

SDS-PAGE was used to separate equal amounts of protein (quantified by Sypro^®^ Ruby stain) or equal volume of EV or WCL samples. Proteins were transferred to the nitrocellulose membrane (Thermo scientific), blocked with 5% skim milk and probed with the following antibodies: TSG101 (BD Transduction Laboratories: catalog number—612696), CD63 (Bio-Rad: catalog number—MCA2042GA), p16 (Cell Signaling Technologies^®:^ catalog number—2407S), GSK3-β (Gene Tex: catalog number—GTX111192), Vimentin (Cell Signaling Technologies^®:^ catalog number—5741S), GAPDH (Cell Signaling Technologies^®:^ catalog number—5174S), Twist (Abcam^:^ catalog number—ab50857), Snail (Cell Signaling Technologies^®:^ catalog number—3879S), p-MAPK (Cell Signaling Technologies^®:^ catalog number—9101S), p-STAT (Cell Signaling Technologies^®:^ catalog number—9134S), p62 (Cell Signaling Technologies^®:^ catalog number—5114S), p53 (Cell Signaling Technologies^®:^ catalog number – 2524S) and β-actin (Cell Signaling Technology^®^ catalog number—4970). Fluorescent conjugated rabbit and mouse secondary antibodies were used and the protein bands were visualized using ODYSSEY CLx (LI-COR^®^).

### Transmission electron microscopy

EV samples (0.2 µg/µL) were examined in a JEM-2010 transmission electron microscope (JEOL, 80 kV) or Tecnai TF30 transmission electron microscope (FEI, 300 kV) as described previously^43^.

### Nanoparticle tracking analysis (NTA)

NTA was performed using NanoSight NS300 machine (Malvern Instruments, Malvern, UK) equipped with a sample chamber of 405 nm laser. Samples (1–2 μg/mL) were disaggregated using a needle and syringe before injecting into the NanoSight sample cubicle (30 frames per second). The parameters maintained during the experiments include camera level at 7, detection threshold at 10, flowrate at 50, and the temperature was maintained at 25 °C. Data analysis was done using the NanoSight NTA 3.2 software.

### EVs stability experiment

Milk-derived EVs and LIM1215-derived EVs were incubated with 1 mM of EGTA (Bio Basic) and CaCl_2_ (Sigma-Aldrich), respectively for 30 min. They were acidified (pH = 2, 15 min) and boiled (105 °C, 15 min) and further subjected to ultracentrifugation at 100,000 *g* for 1 h at 4 °C (Beckman Coulter: TLA-55 rotor). The obtained EV pellet was subjected to western blotting (equal volume loaded) and NTA analysis.

### In gel digestion

Equal amounts of proteins obtained from EV (30 µg) or liver lysate samples (30 µg) were loaded onto precast NuPAGE^®^ 4–12% Bis-Tris gels in 1× MES SDS running buffer. Gels were run at a constant voltage of 150 V followed by visualization of proteins with Coomassie stain (Bio-Rad). Gel bands (20) were excised and subjected to in gel reduction, alkylation, and trypsinization as described previously^44,45^. Briefly, gel bands were reduced with 10 mM DTT (Bio-Rad), alkylated with 25 mM iodoacetamide (Sigma) and digested overnight at 37 °C with 150 ng of sequencing grade trypsin (Promega). The tryptic peptides were extracted by 0.1% trifluoroacetic acid in 50% (w/v) acetonitrile.

### LC–MS/MS

Samples were analyzed by LC–MS/MS using Q-Exactive plus and Fusion Lumos Orbitrap mass spectrometer (Thermo Scientific), both fitted with nanoflow reversed-phase-HPLC (Ultimate 3000 RSLC, Dionex). The nano-HPLC system was equipped with an Acclaim Pepmap nano-trap column (Dionex—C18, 100 Å, 75 μm × 2 cm) and an Acclaim Pepmap RSLC analytical column (Dionex—C18, 100 Å, 75 μm × 50 cm). Typically, for each LC-MS/MS experiment, 1 μL of the peptide mix was loaded onto the enrichment (trap) column at an isocratic flow of 5 μL/min of 3% CH_3_CN containing 0.1% formic acid for 5 min before the enrichment column is switched in-line with the analytical column. The eluents used for the LC were 0.1% (v/v) formic acid (solvent A) and 100% CH_3_CN/0.1% (v/v) formic acid. The gradient used was 3% B to 25% B for 23 min, 25% B to 40% B in 2 min, 40% B to 85% B in 2 min, and maintained at 85% B for 2 min before equilibration for 10 min at 3% B prior to the next injection. All spectra were acquired in positive mode with full scan MS spectra scanning from *m*/*z* 300–1650 in the FT mode at 70 000 (QE) and 120,000 (Lumos) resolution. Lockmass of 445.12003 *m*/*z* was used for both instruments. For MSMS on the Lumos, the “top speed” acquisition method mode (3 s cycle time) on the most intense precursor was used whereby peptide ions with charge states ≥2 were isolated with isolation window of 1.6 *m*/*z* and fragmented with HCD using normalized collision energy of 35. For MSMS on the QE plus, the 15 most intense peptide ions with charge states ≥2 were isolated with isolation window of 1.6 *m*/z and fragmented by HCD with normalized collision energy of 35. Dynamic exclusion of 30 s was applied.

### Database searching, protein identification, and label-free spectral counting

Peak lists were extracted from raw mass spectrometry into the Mascot Generic File Format (MGF) using MsConvert with peak picking. The MGF files were then searched using X! Tandem (Sledgehammer edition version 2013.09.01.1) against a mouse and/or bovine or NR protein database. Search parameters used were fixed modification (carboamidomethylation of cysteine; +57 Da), variable modifications (oxidation of methionine; +16 Da and N-terminal acetylation; +42 Da), two missed tryptic cleavages, 20 ppm peptide mass tolerance, and 0.6 Da fragment ion mass tolerance. Proteins were quantified using the Normalized Spectral Abundance Factor method^46^.

### Proteotypic peptides

Peptides uniquely identified in liver tissue of mice orally administered with milk-derived EVs were subjected to BLAST against the NR protein database. Peptides unique to Bovidae and not 100% homologous to mouse and other species were filtered.

### Functional enrichment analysis

Functional enrichment analysis was performed using FunRich tool^26^. The heatmap, Venn diagram, and gene ontology analysis were obtained from FunRich. Statistical analysis for gene set enrichment was performed using inbuilt analysis tools in FunRich.

### Small RNA extraction

The total RNA including miRNA from samples were extracted using a column-based miRNeasy kit (Qiagen, Chadstone, VIC, Australia) with the following modifications to the manufacturer’s instructions. TRIzol-LS (Life Technologies), instead of QIAzol, was added to the EV samples and subsequently vortexed. After incubation, chloroform was added to the homogenate and the manufacturer’s protocol of the miRNeasy kit was resumed to isolate RNA sized between 18 nt and above. The quantity and quality of the small RNA extractions were determined using a BioSpec-nano (Shimadzu, Rowville, VIC, Australia) and Agilent Bioanalyser 2100 with a small RNA assay Chip for cell-free exosomal small RNA, and a RNA Nano 6000 assay Chip for cellular RNA (Agilent Technologies, Mulgrave, VIC, Australia).

### Small RNA library construction and sequencing

Small RNA library preparation was performed using the Ion Total RNA-Seq kit v2 (Life Technologies) for both EV samples. For each individual library, RNA was ligated to adapters containing a unique index barcode (Ion Xpress™ RNA-Seq Barcode 1–16 Kit, Life Technologies) to allow libraries to be pooled during Ion-Torrent sequencing (Life Technologies). All libraries were constructed according to manufacturer’s protocol. Briefly, RNA samples were reverse transcribed to cDNA using adapter-specific primers. Using the Magnetic Bead Purification Module (Life Technologies), cDNA samples were size-selected from 94 to 200 nt (the length of the small RNA insert including the 3′ and 5′ adapters). PCR amplification was then performed followed by a library clean-up step using nucleic acid beads (Life Technologies). The quality and quantity of each library were determined by Agilent 2100 Bioanalyser using High Sensitivity DNA kit (Agilent Technologies). Equally pooled libraries were clonally amplified onto Ion Sphere™ Particles (ISPs) supplied by the Ion PGM™ Template OT2 200 kit (Life Technologies). ISP templates were produced by using the OneTouch™ 2 Instrument and enrichment system (Life Technologies). ISPs loaded with libraries were sequenced on the Ion-Torrent PGM™ using Ion™ 318 v2 chips (Life Technologies) and the Ion PGM™ 200 Sequencing Kit v2 (Life Technologies).

### Small RNA analysis

IonTorrent generated basecaller bams were converted to fastq files using Picard tool (v1.105). The fastq sequence reads were mapped against Bos Taurus genome (v4.6.1) using Bowtie2 in unpaired fashion. The mapped reads were further assembled into transcripts and its abundance estimated in FPKM (fragments per kilobase of transcript per million fragments mapped) for comparison between the samples using Cufflinks (v2.2.1) suite of tools.

### In vivo biodistribution of EVs

Immunocompetent Balb/c mice (*n* = 3 per group) were utilized to study biodistribution of EVs administered via oral delivery. Milk, yeast, and beer EVs were labeled with near-infrared fluorescent dye DiR (5 µM, PerkinElmer) by incubation at 37 °C for 15 min followed by centrifugation at 100,000 × *g* for 1 h at 4 °C (Beckman Coulter: TLA-55 rotor) to remove unbound dye as described previously^47^. EV pellets were further resuspended in PBS and animals were administered with a single dose of DiR-labeled EVs (25 mg/kg). Mice were further imaged using IVIS Lumina XR-III (Caliper Life Sciences) at 6, 12, 24, and 48 h. In addition, organs were rapidly excised from euthanized animals and subjected to ex vivo imaging. The relative intensities were measured and compared with vehicle (PBS) only control as well as free dye control. EVs were sonicated (settings: 60% amplitude, 6 cycles of 30 s on/off for 3 min with 2 min cooling period between each cycle using Sonicator Vibra-Cell (Sonics & Materials)) and labeled with DiR. Equivalent amount of whole CM (70 μL) was supplemented with 500 µg of labeled EVs and was also compared to WM with free dye by in vivo imaging after oral administration to the mice. The images were further analyzed using Living Image 4.4 software (Caliper Life Sciences). Animal experiments were conducted in accordance with the Australian code of practice for the care and use of animals for scientific purposes and in compliance La Trobe Ethics Committee guidelines (AEC 16–75 and AEC 15–23).

### Senescence β-Galactosidase Assay

Cells were seeded with the density of 2 × 10^5^ in a 12-well plate and grown to over 60% confluency. The cells were washed with 1× PBS. 1× Fixative Solution (Cell Signaling Technologies) was added to each well-containing cells and incubated for 15 min at room temperature. The cells were washed twice with 1× PBS then β-Galactosidase Staining Solution (pH 6.0) (Cell Signaling Technologies) was added. The plate was sealed with parafilm and incubated overnight at 37 °C without CO_2_. β-Galactosidase Staining Solution was removed and 70% (v/v) glycerol was added to each well-containing cells. Images were taken using a IX81 Motorized Inverted Microscope (OLYMPUS^®^) with 10× magnification.

### FACS cell cycle analysis

Cells were seeded at a density of 5 × 10^3^ cells per well in a 24-well plate in 500 µL RPMI 1640 culture medium and allowed to adhere for 3 and 4 days at 37 °C at 5% CO_2_. Cells were then treated with 20 µg/mL of milk-derived EVs and incubated for 72 h. At this time point, cells were scraped and resuspended to collect 200 μL from each well and transferred into a 96-well plate and spun at 300 *g* for 5 min. The supernatant was discarded and the pellet was resuspended in 200 μL of PI-Hypotonic lysis buffer (0.1% (w/v) sodium citrate, 0.1% Triton X 100 (w/v), 50 μg/mL propidium iodide (Sigma Life Science^®^)) in milliQ and incubated overnight at 4 °C. Samples were then subjected to FACS CANTO II (BD Biosciences) and analyzed using FlowJo (TreeStar).

### Cell viability assay

The amount of ATP in treated cells was determined using CellTiter-Glo 2.0 kit (Promega;WI, USA). Two thousand cells per well were seeded in 96-well white plates and were treated with 1 ng/mL of murine TNF-α (Peprotech; NJ, USA), 10 µM of SM-164 (ApexBio; Texas, USA), 10 µM of QVD (R&D systems), 10 µM of Necrostatin-1 (Sigma-Aldrich) or milk-derived EVs (100 µg/mL). After 48 h, 75 μL of CellTiter-Glo was added to each well and the plates were incubated at room temperature for 10 min. Luminescence was recorded using a Spectromax M5 (Molecular Devices; CA, USA).

### Clonogenic assay

SW620 (colorectal cancer) cells were seeded in triplicates in 6-well plates at a density of 1000 cells/well. The cells were treated with 20 µg/mL of milk-derived EVs for 72 h. The cells were further cultured for ~15 days, the colonies were stained with 0.5 % (w/v) crystal violet in 20% (w/v) methanol before counting them.

### Wound healing assay

Cells were seeded and allowed to reach 100% confluence in six-well plates. Pipette tip was then used to scratch the monolayer of cells and the medium was changed to fresh medium (with or without milk-derived EV or 4T1.2 EVs). The 6-well plates were then incubated at 37 °C in 5% CO2. The wound closure was monitored at various time points under the light microscope and the wound area was analyzed using imageJ 1.47 application.

### Establishment of CRISPR/Cas9-mediated knockout cells

CRISPR/Cas9-based ATG5 KO cells were generated as previously described^48^. First, HEK-293T cells were used to produce Cas9 and gRNA (targeting ATG5) vectors containing lentiviral particles. Next, the LIM1215 cells were infected with the viral particles and followed by induction with doxycycline (Sigma-Aldrich). The cells were then subjected to single-cell sorting for GFP and mCherry positive cells using FACS Aria II (BD Biosciences). The obtained clones were further validated by western blotting.

### Establishing tumor xenografts in athymic nude mice

SW620 cells (2.5 × 10^6^) were s.c. injected in 6–8-week-old female BALB/*c*-Fox1nuAusb mice. The mice were daily gavaged (p.o.) with a single dose of RM and CM EVs at a concentration of 25 mg/kg. Control mice were injected with vehicle only (PBS). Tumor size was measured daily using digital calipers and tumor volume was calculated according to the formula ½ (W^2^ × L). Mice were sacrificed when the cumulative primary tumor volume reached 1500 mm^3^. Animal experiments were conducted in accordance with the Australian code of practice for the care and use of animals for scientific purposes and in compliance La Trobe Ethics Committee guidelines (AEC 14–15).

### CD2F1 mice model for cancer-induced weight loss

C-26 cells were gifted by N.J.H. (La Trobe University, Melbourne). Prior (before 5 days) to the implantation of tumor cells, milk-derived EVs were daily (single dose) administered to CD2F1 mice by oral gavaging at a concentration of 25 mg/kg. C-26 cells (5 × 10^6^) were s.c. injected into CD2F1 immunocompetent mice on day 6. Milk-derived EVs were daily (single dose) administered to CD2F1 mice by oral gavaging (p.o.) at a concentration of 25 mg/kg. Tumor measurements were taken using digital calipers and volume calculated with the formula ½(W^2^ × L). The weight of the mice was also monitored regularly. For pair feeding, the food intake of ‘control’ tumor group for 24 h was monitored and the PF group was provided with the same amount of food for the next 24 h. Experimental endpoints were determined by monitoring the body conditions of the mice like overnight weight loss >10% of body weight, or hunching posture, signs of pain or when the cumulative primary tumor volume reached 1500 mm^3.^ Animal experiments were conducted in accordance with the Australian code of practice for the care and use of animals for scientific purposes and in compliance La Trobe Ethics Committee guidelines (AEC 14–15).

### Intrasplenic injections of cancer cells and in vivo imaging

For pancreatic metastasis study, intrasplenic injections were performed with KPC (pancreatic cancer) cells (5 × 10^5^) expressing a GFP-firefly Luciferase construct in BALB/*c*-Fox1nuAusb mice during open laparotomy (anesthetized with isoflurane 3 L, O_2_ 1 L/min, vacuum was used constantly to remove excess of O_2_) as previously described^31^. Metastatic spread was monitored longitudinally using IVIS imaging of luciferase signal (intraperitoneal. injection of 10 μL/g d-Luciferin (Gold Biotechnology)) on day 7, day 10, and day 12 following intrasplenic injections (IVIS Spectrum, PerkinElmer). Mice were subjected to 5 days of pre-treatment with milk-derived EVs before intrasplenic injections and were administered with milk-derived EVs daily after intrasplenic injections and until endpoint. On day 12, mice were sacrificed, organs were collected and metastatic burden was measured by IVIS imaging in liver and quantification of visible liver metastases at the surface of the organ. Assessment of metastatic burden was also performed by quantifying the number of metastases in H&E sections of the liver, as previously performed^31^. Animal experiments were conducted in accordance with the Australian code of practice for the care and use of animals for scientific purposes and in compliance with Garvan Ethics Committee guidelines (16/13 protocol).

### Histology and immunostaining of primary tumor tissues of breast cancer model

For histological evaluation of treatment effect, tumors from CM, RM experimental, and PBS control groups were processed for paraffin embedding and sectioned at 7 µm. Immunostaining was performed using paraffin sections essentially as described with *n* = 3 mice per group^49^. Following antigen unmasking in citrate solution (113.93 mM Na_3_C_6_H_5_O_7_, pH 6), incubation with combined primary antibodies including polyclonal anti-Ki67 (at 1:200, Abcam, Cambridge, UK) and monoclonal anti-vimentin (at 1:400, Sigma-Aldrich, St Louis, Mo.) was performed overnight at 4 °C. This was followed by amplification of signals from anti-Ki67 with biotinylated anti-rabbit Ig (Vector Laboratories). Detection was performed with streptavidin-Alexa 488 and anti-mouse Ig-Alexa 594 (Thermo Fisher Scientific, Waltham, MA). Nuclei were stained with 0.001% 4′, 6 diamidino-2-phenylindole (DAPI). Images were captured on confocal microscope Zeiss LSM510 under 40X/1.3 oil DIC M27 objective and images processed with the Zen software (Zeiss, Welwyn Garden City, and UK) and ImageJ application was used to analyze and generate images.

### Immunohistochemistry and quantification of liver metastases

After euthanasia, tissues were removed and fixed in 10% buffered formalin and embedded in paraffin. Sections were stained with H&E stain on a Leica autostainer. Total number of liver metastases were quantified on serial sections (5 sections per organ with a 100 μm step). The area covered by the whole liver tissue was measured with ImageJ (US National Institutes of Health). For analysis, individual metastases were counted and quantified.

### Orthotopic injection of tumor cell and ex vivo imaging

4T1.2 (murine breast cancer) cells (1 × 10^5^) expressing luciferase (Luc2) reporter gene were injected into the 4th mammary gland of 8- to 12-week-old female BALB/c mice. Primary tumor size was measured regularly using electronic calipers, and tumor volume (mm^3^) was calculated as ½(W^2^ × L). If indicated, primary tumors were resected when the tumor volume reached 150 mm^3^ at 10 days after tumor cell inoculation. Mice were sacrificed upon signs of metastatic distress or when the cumulative primary tumor volume reached 1500 mm^3^. The extent of metastasis in particular organs was assessed using quantitative real-time PCR (qPCR). In addition, lungs were rapidly excised from euthanized animals and subjected to ex vivo imaging using an IVIS Lumina XR-III (Caliper Life Sciences). Bioluminescent intensity 12 min after i.p. injection of 1.5 mg d-Luciferin (Gold Biotechnology) was normalized between all images in a group using Living Image 4.4 software (Caliper Life Sciences). Metastatic burden was also measured by quantification of lung metastases visible at the surface of the organ. Animal experiments were conducted in accordance with the Australian code of practice for the care and use of animals for scientific purposes and in compliance La Trobe Ethics Committee guidelines (AEC 16–75).

### Quantification of metastatic burden

Duplex qPCR was used to quantify metastatic burden as previously described^50^ by comparing the ratio of *mCherry* (present in tumor cells) and *vimentin* (present in all cells) sequences in genomic DNA preparations from homogenized and proteinase-K (100 μg/mL, Merck) digested lungs. Primers were as follows: *mCherry* fwd: 5′-GACCACCTACAAGGCCAAGAAG-3′, rev: 5′-AGGTGATGTCCAACTTGATGTTGA-3′, hydrolysis probe: 5′FAM-CAGCTGCCCGGCGCCTACA-3′TAMRA and *vimentin* fwd: 5′-AGCTGCTAACTACCAGGACACTATTG-3′, rev: 5′-CGAAGGTGACGAGCCATCTC-3′, hydrolysis probe: 5′VIC-CCTTCATGTTTTGGATCTCATCCTGCAGG-3′TAMRATumor metastatic burden (arbitrary units) was based on the quantification cycle (Cq) for mCherry relative to vimentin and displayed as relative tumor burden (RTB) using the following equation:

$${\rm{RTB}}=10,000/({2}^{\Delta {C_{\rm{q}}}})$$

where ∆*C*_q_ = *C*_q_ (target gene) − *C*_q_ (control)

### Flow cytometric analysis of immune cell population

Blood obtained from cardiac bleeds was used to profile circulating immune cells after red blood cell lysis (155 mM NH4Cl, 10 mM KHCO_3_, 0.1 mM EDTA, pH 7.3). Cells were stained with the following antibodies: CD8a-PE-Cy7 (53-6.7), CD4-APC-Cy7 (GK1.5), CD69-APC (H1.2F3), CD279-PE (J43), Ly6G-BV421 (1A8), B220 Pe-Cy5, CD103 bv510, CD11b BV605, CD11c Percpm, CD206 APC, CD25 APC, CD27 A-Cy7, CD4 A-Cy7, CD4 Pe-Cy7, CD44 FITC, CD62L BV450, CD69 FITC, CD69 Pe-Cy7, CD8 A-Cy7, CD8 APC, CD8 Pe-Cy5, CD8 Pe-Cy7, CD80 PE, F4/80 Pe-Cy7, FOXP3 FITC, H2-Kd FITC, IFNAR1 PE, IFNg PE, Ly6C-APC, Ly6G BV711, MHC-II BV650, NKG2D Pe-Cy7, NKp46 BV421, PD1 PE, PDL1 BV421, Rat IgG2a FITC, TCRbeta BV510, TNFa FITC (all from BD Biosciences) and Ly6C-APC (HK1.4) (Biolegend). Primary tumors were mechanically and enzymatically digested with 1 mg/mL collagenase I (Sigma) and 30 μg/mL DNAse I (Sigma) at 37 °C to obtain a single cell suspension before red blood cell lysis. Analysis of tumor-infiltrating lymphocytes was done as above. Analysis of immune cell populations was performed by flow cytometry using a FACSCanto II (BD Biosciences, USA). Data was analyzed using Flowjo software (TreeStar, USA).

### Comet assay

Comet assay was performed as per manufacturer’s instructions (ab238544—Comet Assay Kit). Briefly, comet agarose was pipetted onto the comet slide to form the baser layer. A single-cell suspension of cells (100,000 untreated, 1 μM doxorubicin treated (48 h), 100 μg/mL CM EVs treated and 200 μg/mL CM EVs treated) were combined with comet agarose at 37 °C. The agarose and cell mixture were then added onto the top of the base layer. Then slides were then incubated with neutral lysis buffer and electrophoresis was performed under neutral conditions. The cells were then stained with DNA dye and analyzed under the fluorescent microscope. Here, 1 μM doxorubicin was used as the positive control.

### Reporting summary

Further information on research design is available in the Nature Research Reporting Summary linked to this article.

## Supplementary information

Supplementary information

Description of Additional Supplementary Files

Dataset 1

Dataset 2

Dataset 3

Dataset 4

Dataset 5

Reporting Summary

## Data Availability

Small RNA sequencing data of milk-derived EVs has been deposited with links to BioProject accession number PRJNA702232 in the NCBI BioProject database. Proteomics data of milk-derived EVs, breast cancer cells, and liver tissue have been deposited to the ProteomeXchange Consortium via the PRIDE partner repository (PXD024739, PXD024762, PXD024821). All additional experimental data are available from the corresponding author on request. Source data are provided with this paper.

## Source data

Source Data

## References

[1] Zhang L (2012). Exogenous plant MIR168a specifically targets mammalian LDLRAP1: evidence of cross-kingdom regulation by microRNA. Cell Res..

[2] Samuel, M., Bleackley, M., Anderson, M. & Mathivanan, S. Extracellular vesicles including exosomes in cross kingdom regulation: a viewpoint from plant-fungal interactions. *Front. Plant Sci.* **6**, 10.3389/fpls.2015.00766 (2015). 10.3389/fpls.2015.00766 PMC4585280 26442078

[3] Chin AR (2016). Cross-kingdom inhibition of breast cancer growth by plant miR159. Cell Res..

[4] Vaucheret H, Chupeau Y (2012). Ingested plant miRNAs regulate gene expression in animals. Cell Res..

[5] Witwer KW, Hirschi KD (2014). Transfer and functional consequences of dietary microRNAs in vertebrates: Concepts in search of corroboration. BioEssays.

[6] Dickinson B (2013). Lack of detectable oral bioavailability of plant microRNAs after feeding in mice. Nat. Biotechnol..

[7] Snow JW, Hale AE, Isaacs SK, Baggish AL, Chan SY (2013). Ineffective delivery of diet-derived microRNAs to recipient animal organisms. RNA Biol..

[8] Baier SR, Nguyen C, Xie F, Wood JR, Zempleni J (2014). MicroRNAs are absorbed in biologically meaningful amounts from nutritionally relevant doses of cow milk and affect gene expression in peripheral blood mononuclear cells, HEK-293 kidney cell cultures, and mouse livers. J. Nutr..

[9] Izumi H (2012). Bovine milk contains microRNA and messenger RNA that are stable under degradative conditions. J. Dairy Sci..

[10] Dever JT (2015). Survival and diversity of human homologous dietary MicroRNAs iN Conventionally Cooked Top Sirloin and Dried Bovine Tissue Extracts. PLoS ONE.

[11] Samuel, M. et al. Bovine milk-derived exosomes from colostrum are enriched with proteins implicated in immune response and growth. *Scie. Rep.* **7**, 10.1038/s41598-017-06288-8 (2017). 10.1038/s41598-017-06288-8 PMC5517456 28725021

[12] Sanwlani, R., Fonseka, P., Chitti, S. V. & Mathivanan, S. Milk-derived extracellular vesicles in inter-organism, cross-species communication and drug delivery. *Proteomes* **8**, 10.3390/proteomes8020011 (2020). 10.3390/proteomes8020011 PMC7356197 32414045

[13] Munagala R, Aqil F, Jeyabalan J, Gupta RC (2016). Bovine milk-derived exosomes for drug delivery. Cancer Lett..

[14] van Herwijnen MJC (2016). Comprehensive proteomic analysis of human milk-derived extracellular vesicles unveils a novel functional proteome distinct from other milk components. Mol. Cell. Proteom..

[15] Kroenke CH, Kwan ML, Sweeney C, Castillo A, Caan BJ (2013). High- and low-fat dairy intake, recurrence, and mortality after breast cancer diagnosis. J. Natl Cancer Inst..

[16] Wiley AS (2012). Cow milk consumption, insulin‐like growth factor‐I, and human biology: a life history approach. Am. J. Hum. Biol..

[17] Benmoussa A (2016). Commercial dairy cow milk microRNAs resist digestion under simulated gastrointestinal tract conditions. J. Nutr..

[18] Anand, S., Samuel, M., Kumar, S. & Mathivanan, S. Ticket to a bubble ride: cargo sorting into exosomes and extracellular vesicles. *Biochim. Biophys. Acta Proteins Proteom*. 10.1016/j.bbapap.2019.02.005 (2019). 10.1016/j.bbapap.2019.02.005 30822540

[19] Wolf T, Baier SR, Zempleni J (2015). The intestinal transport of bovine milk exosomes is mediated by endocytosis in human colon carcinoma Caco-2 cells and rat small intestinal IEC-6 cells. J. Nutr..

[20] Gangoda L, Boukouris S, Liem M, Kalra H, Mathivanan S (2015). Extracellular vesicles including exosomes are mediators of signal transduction: are they protective or pathogenic?. Proteomics.

[21] Lotvall J (2014). Minimal experimental requirements for definition of extracellular vesicles and their functions: a position statement from the International Society for Extracellular Vesicles. J. Extracell. Vesicles.

[22] Reinhardt TA, Lippolis JD, Nonnecke BJ, Sacco RE (2012). Bovine milk exosome proteome. J. Proteom..

[23] Le A, Barton LD, Sanders JT, Zhang Q (2010). Exploration of bovine milk proteome in colostral and mature whey using an ion-exchange approach. J. Proteome Res..

[24] Pieters, B. C. H. et al. Commercial cow milk contains physically stable extracellular vesicles expressing immunoregulatory TGF-beta. *PLoS ONE* **10**, e0121123 (2015). 10.1371/journal.pone.0121123 PMC4379073 25822997

[25] Pathan M (2019). Vesiclepedia 2019: a compendium of RNA, proteins, lipids and metabolites in extracellular vesicles. Nucleic Acids Res..

[26] Pathan M (2017). A novel community driven software for functional enrichment analysis of extracellular vesicles data. J. Extracell. Vesicles.

[27] Keerthikumar S, Mathivanan S (2017). Proteotypic peptides and their applications. Methods Mol. Biol..

[28] Marangoni F (2019). Cow’s milk consumption and health: a health professional’s guide. J. Am. Coll. Nutr..

[29] Somiya M, Yoshioka Y, Ochiya T (2018). Biocompatibility of highly purified bovine milk-derived extracellular vesicles. J. Extracell. Vesicles.

[30] Chitti SV, Fonseka P, Mathivanan S (2018). Emerging role of extracellular vesicles in mediating cancer cachexia. Biochem. Soc. Trans..

[31] Chin, V. T. et al. Transient tissue priming via ROCK inhibition uncouples pancreatic cancer progression, sensitivity to chemotherapy, and metastasis. *Sci. Transl. Med.* **9**, eaai8504 (2017). 10.1126/scitranslmed.aai8504 PMC5777504 28381539

[32] Fonseka, P., Pathan, M., Chitti, S. V., Kang, T. & Mathivanan, S. FunRich enables enrichment analysis of OMICs datasets. *J. Mol. Biol*. 166747, 10.1016/j.jmb.2020.166747 (2020). 10.1016/j.jmb.2020.166747 33310018

[33] Peinado H (2012). Melanoma exosomes educate bone marrow progenitor cells toward a pro-metastatic phenotype through MET. Nat. Med..

[34] Faget DV, Ren Q, Stewart SA (2019). Unmasking senescence: context-dependent effects of SASP in cancer. Nat. Rev. Cancer.

[35] Takasugi M (2017). Small extracellular vesicles secreted from senescent cells promote cancer cell proliferation through EphA2. Nat. Commun..

[36] Takahashi A (2017). Exosomes maintain cellular homeostasis by excreting harmful DNA from cells. Nat. Commun..

[37] Rodier F, Campisi J (2011). Four faces of cellular senescence. J. Cell Biol..

[38] Zempleni J (2017). Milk exosomes: beyond dietary microRNAs. Genes Nutr..

[39] Fonseka P, Chitti SV, Sanwlani R, Mathivanan S (2021). Sulfisoxazole does not inhibit the secretion of small extracellular vesicles. Nat. Commun..

[40] Tulkens J (2020). Increased levels of systemic LPS-positive bacterial extracellular vesicles in patients with intestinal barrier dysfunction. Gut.

[41] Kamerkar S (2017). Exosomes facilitate therapeutic targeting of oncogenic KRAS in pancreatic cancer. Nature.

[42] Lehmann BD (2008). Senescence-associated exosome release from human prostate cancer cells. Cancer Res..

[43] Keerthikumar S (2015). Proteogenomic analysis reveals exosomes are more oncogenic than ectosomes. Oncotarget.

[44] Mathivanan S, Ji H, Tauro BJ, Chen YS, Simpson RJ (2012). Identifying mutated proteins secreted by colon cancer cell lines using mass spectrometry. J. Proteomics.

[45] Fonseka P (2019). Exosomes from N-Myc amplified neuroblastoma cells induce migration and confer chemoresistance to non-N-Myc amplified cells: implications of intra-tumor heterogeneity. J. Extracell. Vesicles.

[46] Paoletti AC (2006). Quantitative proteomic analysis of distinct mammalian Mediator complexes using normalized spectral abundance factors. Proc. Natl Acad. Sci. USA.

[47] Kalra H (2019). Extracellular vesicles containing oncogenic mutant beta-catenin activate Wnt signalling pathway in the recipient cells. J. Extracell. Vesicles.

[48] Kueh AJ, Herold MJ (2016). Using CRISPR/Cas9 technology for manipulating cell death regulators. Methods Mol. Biol..

[49] Pham H (2011). Experimental autoimmune encephalomyelitis (EAE) IN C57Bl/6 mice is not associated with astrogliosis. J. Neuroimmunol..

[50] Eckhardt BL (2005). Genomic analysis of a spontaneous model of breast cancer metastasis to bone reveals a role for the extracellular matrix. Mol. Cancer Res..

