# Supplementary material for: Oral administration of bovine milk-derived extracellular vesicles induces senescence in the primary tumor but accelerates cancer metastasis
Source: Nat Commun. 2021 Jun 24;12:3950. doi: 10.1038/s41467-021-24273-8 (PMC8225634; doi:10.1038/s41467-021-24273-8)

Supplementary information

Oral administration of bovine milk-derived extracellular vesicles induces senescence in the primary tumor but accelerates cancer metastasis

Monisha Samuel[1], Pamali Fonseka[2], Rahul Sanwlani[2], Lahiru Gangoda[2], Sing Ho Chee[2], Shivakumar Keerthikumar[2,3,4], Alex Spurling[2], Sai V. Chitti[2], Damien Zanker[2], Ching-Seng Ang[5], Ishara Atukorala[2], Taeyoung Kang[2], Sanjay Shahi[2], Akbar L Marzan[2], Christina Nedeva[2], Claire Vennin[6], Morghan C. Lucas[6], Lesley Cheng[2], David Herrmann[6], Mohashin Pathan[2], David Chisanga[2], Sean C. Warren[6], Kening Zhao[2], Nidhi Abraham[2], Sushma Anand[2], Stephanie Boukouris[2], Christopher G. Adda[2], Lanzhou Jiang[2], Tanmay M. Shekhar[2], Nikola Baschuk[2], Christine J. Hawkins[2], Amelia J. Johnston[2], Jacqueline Monique Orian[2], Nicholas J. Hoogenraad[2], Ivan K. Poon[2], Andrew F. Hill[2], Markandeya Jois[1], Paul Timpson[6], Belinda S. Parker[2] and Suresh Mathivanan[2,#]

[1]Department of Physiology, Anatomy and Microbiology, School of Life Sciences, La Trobe University, Bundoora, Victoria, 3086, Australia.

[2]Department of Biochemistry and Genetics, La Trobe Institute for Molecular Science, La Trobe University, Melbourne, Victoria, 3086, Australia.

[3]Cancer Research Division, Peter MacCallum Cancer Centre, Melbourne, VIC 3000, Australia.

[4]Sir Peter MacCallum Department of Oncology, University of Melbourne, VIC 3010, Australia.

[5]Bio21 Institute, University of Melbourne, Victoria, 3010, Australia.

[6]Garvan Institute of Medical Research, The Kinghorn Cancer Centre & St Vincent's Clinical School, Faculty of Medicine, University of New South Wales, Sydney, New South Wales, 2010, Australia.

[#]To whom correspondence should be addressed:

Professor Suresh Mathivanan

Department of Biochemistry and Genetics,

La Trobe Institute for Molecular Science,

La Trobe University,

Bundoora, Victoria 3086, Australia
Tel: +61 03 9479 2565
Email: S.Mathivanan@latrobe.edu.au

Materials

REAGENT or RESOURCE	SOURCE	IDENTIFIER
**Primary Antibodies (Western)**		
TSG101	BD Transduction Laboratories	#612696
p16	Cell Signaling Technologies®	#2407S, #80772
Vimentin	Cell Signaling Technologies®	#5741S
GAPDH	Cell Signaling Technologies®	#5174S
Twist	Abcam	#ab50857
Snail	Cell Signaling Technologies®	#3879S
GSK3-β	Gene Tex	#GTX111192
P-MAPK	Cell Signaling Technologies®	#9101S
MAPK	Cell Signaling Technologies®	#9102S
P-Stat3	Cell Signaling Technologies®	#9134S
Stat3	Cell Signaling Technologies®	#4904S
p62	Cell Signaling Technologies®	#2524S
p53	Cell Signaling Technologies®	#5114S
CD63	BIO-RAD	#MCA2042GA
Ki67	Abcam	#ab15580
CD8a-PE-Cy7 (53-6.7)	BD Biosciences	#552877
CD4-APC-Cy7 (GK1.5)	BD Biosciences	#552051
CD69-APC (H1.2F3)	BD Biosciences	#560689
CD279-PE (J43)	BD Biosciences	#551892
Ly6G-BV421 (1A8)	BD Biosciences	#551461
Ly6C-APC (HK1.4)	Biolegend	#128016
β-actin	Cell Signaling Technologies®	#4970
Caesin	Abcam	#ab166596
ATG5	Cell Signaling Technologies®	#12994S
MMP2	Cell Signaling Technologies®	#13132S

**Secondary Antibodies (Western)**		
Anti-mouse IgG-IRDye® 800CW	LI-COR®	926-32210
Anti-rabbit IgG-IRDye® 680RD	LI-COR®	926-68071
**Primary Antibodies (Immunohistochemistry)**		
Eosin Y solution	Sigma Aldrich	#HT110332
Ki67	Abcam	#ab15580
Vimentin	Sigma Aldrich	#V6630
**Biological Samples**		
Raw milk	Australia DEPI Ellinbank	N/A
Commercial milk	Australian local grocery store	N/A
**Chemicals, Peptides and Recombinant Proteins**		
OptiPrep™ Density Gradient medium	Sigma Life Science®	#D1556
Sucrose	Sigma Life Science®	#S0389
Tris	Astral	#BIO3094T
Sypro® Ruby	Thermo Fisher Scientific	#S12000
EGTA	Bio Basic	#ED0077
$CaCl_2$	Sigma Aldrich	#449709
DTT	Bio-Rad	#1610611
Iodoacetamide	Ameresco	#M216
Propidium Iodide	Sigma Life Science®	#P4170
Trifluoroacetic acid	Thermo Fisher Scientific	#28904
Acetonitrile	Honeywell	#34967
TRIzol-LS	Life Technologies	#10296028
Proteinase-K	Merck	#124568
DiR	PerkinElmer	#125964
BSA	Sigma Aldrich	#A4612

Sodium citrate	Calbiochem®	#567446
Collagenase I	Sigma Life Science®	#C0130
D-luciferin	Gold Biotechnology	#LUCK-100
DNAse I	Sigma Life Science®	#AMPD1
Sequence Grade Modified Trypsin	Promega	#V511A
Crystal violet	Sigma Aldrich	#C6158
murine TNF-α	Peprotech	#315-01A
SM-164	ApexBio	#A8815
Necrostatin-1	Sigma Aldrich	#N9037-10MG
QVD	R&D Systems	#OPH001-01M
Bafilomycin A1	Sigma Aldrich	#B1793
**Commercial Assays**		
miRNeasy kit	Qiagen	#217004
Ion Total RNA-Seq kit v2	Life Technologies	#4475936
Magnetic Bead Purification Module	Life Technologies	#4475486
Ion PGM™ 200 Sequencing Kit v2	Life Technologies	#4482006
Ion PGM™ Template OT2 200 kit	Life Technologies	#4480974
High Sensitivity DNA kit	Agilent Technologies	#5067-4626
CellTiter-Glo 2.0 kit	Promega	#G9241
β-Galactosidase Staining kit	Cell Signaling Technologies®	#9860S
**Cell Lines**		
4T1.2	Gifted by B Parker Lab	N/A
KPC	Gifted by P Timpson Lab	N/A
SW620	Gifted by J Mariadason Lab	N/A
LIM1215	Gifted by J Mariadason Lab	N/A
C26	Gifted by N Hoogenraad Lab	N/A
MCF7	ATCC	N/A
**Organisms/Strains**		

Balb/c-fox1nuausb	Animal Resources Centre, WA	N/A
Balb/c	Animal Resources Centre, WA	N/A
CD2F1	Gifted by N Hoogenraad Lab	N/A
**Software and Algorithms**		
ImageJ	Open source	
Adobe Illustrator	Adobe Illustrator	
FunRich	FunRich	
Graphpad Prism	GraphPad Software	
FlowJo	FlowJo	

Primer name	Sequence (5’ – 3’)	Melting temperature
**mCherry Forward**	GACCACCTACAAGGCCAAGAAG	57.7
**mCherry Reverse**	AGGTGATGTCCAACTTGATGTTGA	56.5
**hydrolysis probe**	FAM-CAGCTGCCCGGCGCCTACA-TAMRA	65.9
**Vimentin Forward**	AGCTGCTAACTACCAGGACACTATTG	N/A
**Vimentin Reverse**	CGAAGGTGACGAGCCATCTC	N/A
**hydrolysis probe**	VIC-CCTTCATGTTTTGGATCTCATCCTGCAGG-TAMRA	N/A

Supplementary Tables

Supplementary Table 1

List of proteins identified in bovine milk-derived extracellular vesicles

Supplementary Table 2

List of RNA identified in bovine milk-derived extracellular vesicles

Supplementary Table 3

List of bovine proteotypic tryptic peptides

Supplementary Table 4

List of proteins differentially abundant in liver tissue of control and milk EVs treated mice

Supplementary Table 5

List of proteins identified in breast cancer cells treated with and without milk EVs for 72 h

Supplementary Figure 1

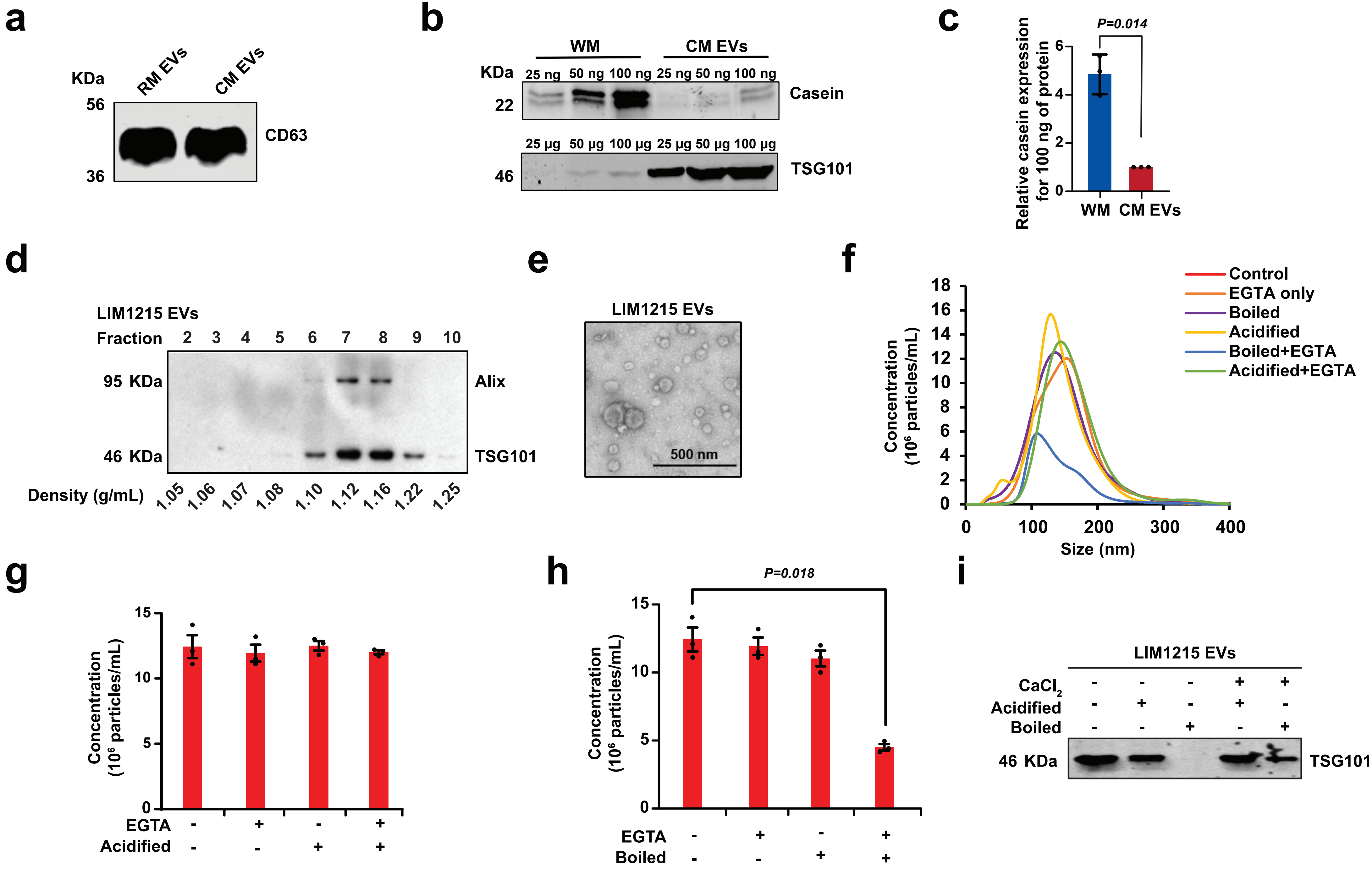

Supplementary Fig. 1

Characterization of milk derived EVs

a, Western blot analysis of EV enriched protein CD63 in milk-derived EV samples. **b,** Western blot analysis of milk abundant protein casein and EV enriched protein TSG101 in WM and CM EVs. **c,** Relative casein abundance in whole milk and CM EVs. **d**, Western blot analysis of EV enriched proteins Alix and TSG101 in LIM1215 colorectal cancer cell-derived EV samples. **e**, TEM images of LIM1215 colorectal cancer cell-derived EVs. **f, g, h,** Stability of EVs was assessed using Nanoparticle Tracking Analysis by comparing particle size and concentration to untreated milk-derived EVs after pre-treatment of EGTA (calcium chelating agent) and further acidifying and boiling. All data are represented as mean ± s.e.m. n=3; Statistical significance was determined by unpaired two-tailed t-test. **i**, Calcium chloride was added to LIM1215-derived EVs prior to acidification or boiling and subjected to Western blot analysis for TSG101.

Supplementary Figure 2

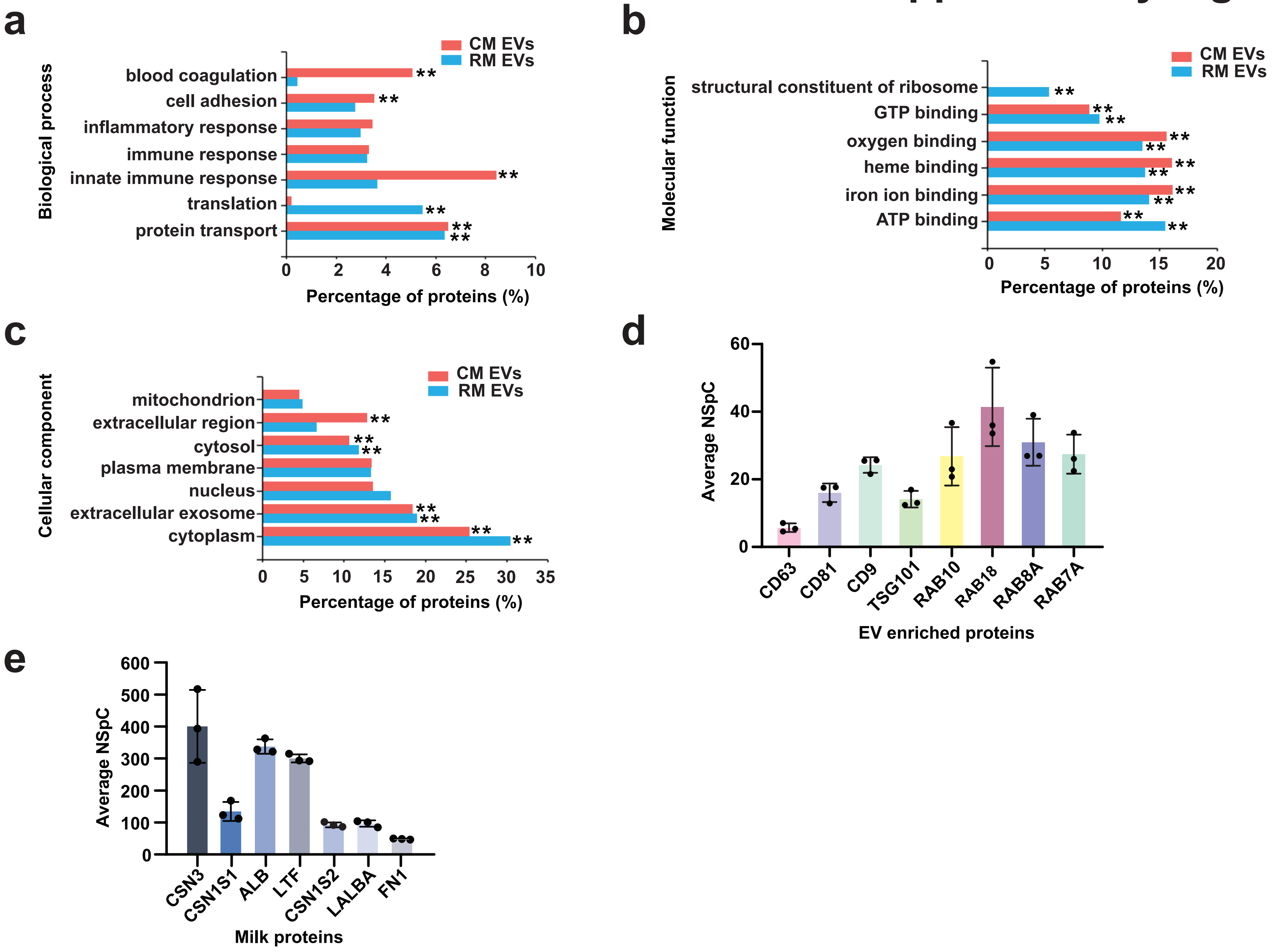

Supplementary Figure 2

Enrichment analysis of proteins identified in milk-derived EVs

a, Biological processes enriched in proteins that are more than 2-fold abundant in RM and CM EVs are displayed (**P<0.01 as determined by hypergeometric test). **b,** Molecular function-based analysis of proteins that are more than 2-fold enriched in RM and CM EVs (**P<0.01 as determined by hypergeometric test). **c,** Cellular component analysis of proteins that are more than 2-fold abundant in RM and CM derived EVs are displayed (**P<0.01 as determined by hypergeometric test). **d,** Normalised spectral counts of EV enriched proteins in CM EVs (*n*=3). **e,** Normalised spectral counts of milk proteins in CM EVs (*n*=3). All data are represented as mean ± s.e.m.

Supplementary Figure 3

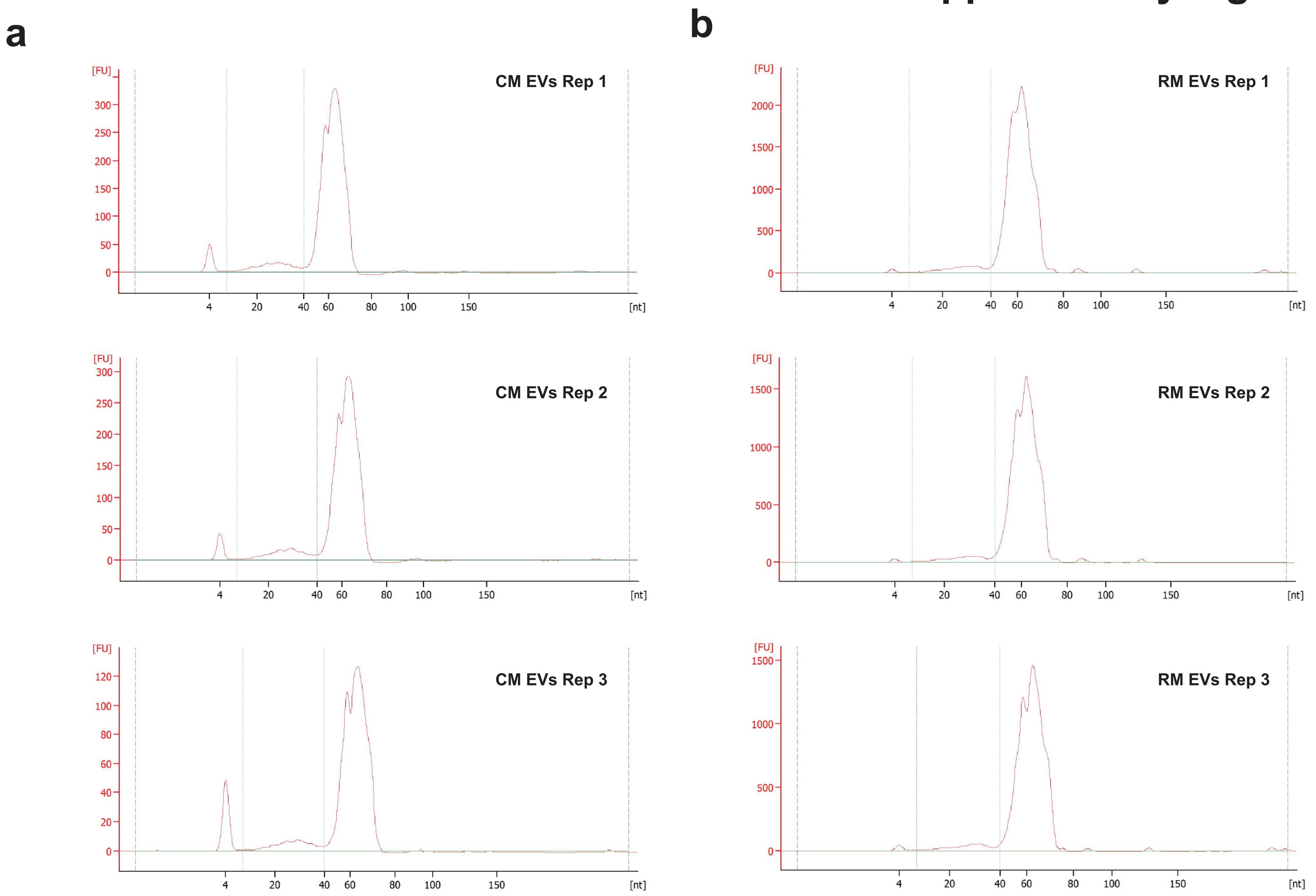

Supplementary Figure 3

Bioanalyzer profile of EVs

a, Bioanalyzer profile of CM EVs – three biological replicates. **b,** Bioanalyzer profile of RM EVs – three biological replicates.

Supplementary Figure 4

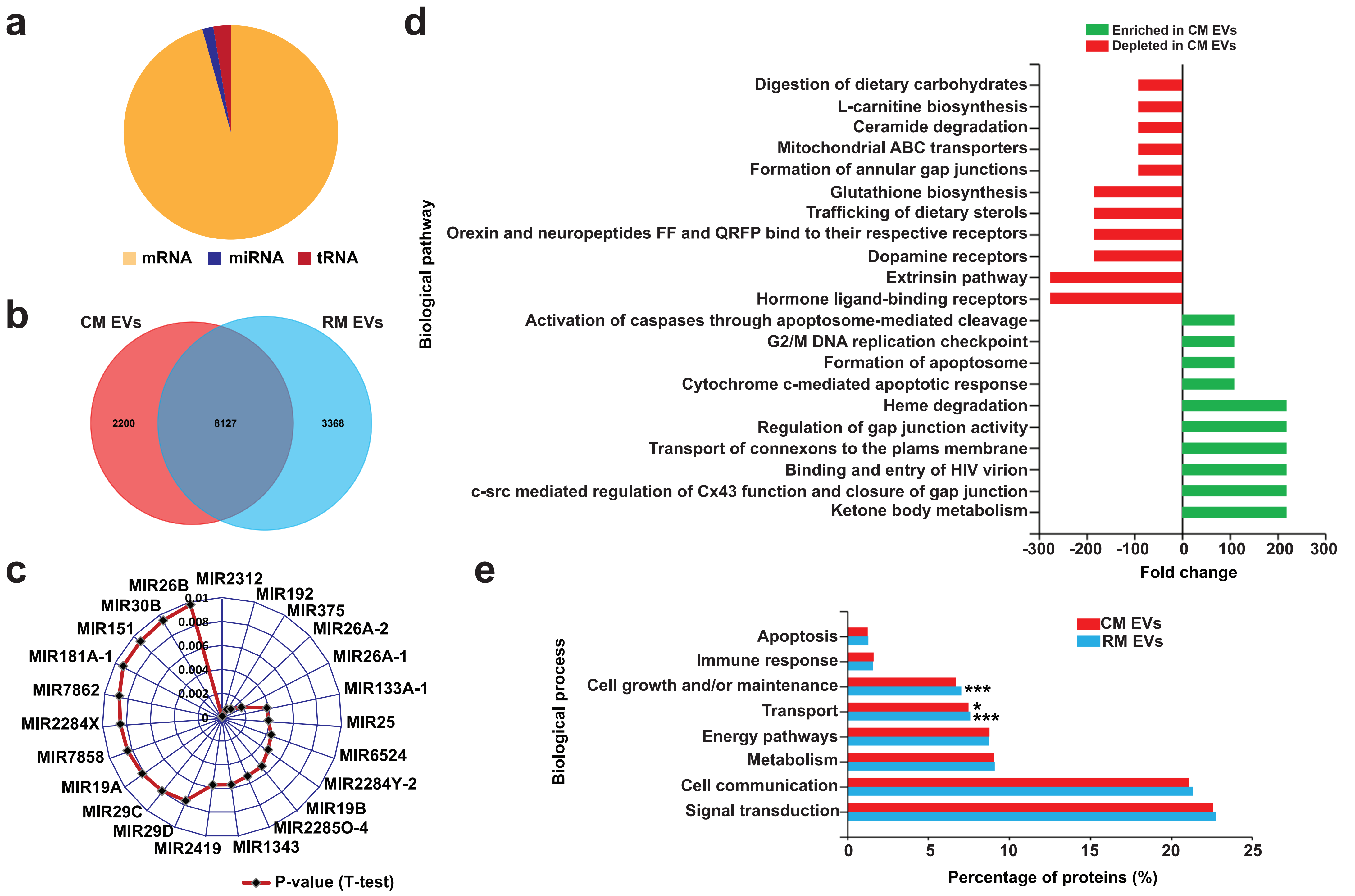

Supplementary Figure 4

Transcriptomic profile of milk-derived EVs

a, Pie chart of RNA distribution in milk-derived EVs. **b,** Venn diagram of RNA identified in all three replicates of CM and RM EVs. **c,** Plot representing significant miRNAs detected in RM or CM EVs (P-value determined by unpaired two-tailed t-test). **d,** Enrichment or depletion of RNA implicated in biological pathways in CM EVs compared to RM EVs is depicted. **e,** Biological process-based analysis of RNAs that are more than 2-fold enriched in RM and CM EV (*P < 0.05, **P<0.01; ***P<0.001 as determined by hypergeometric test).

Supplementary Figure 5

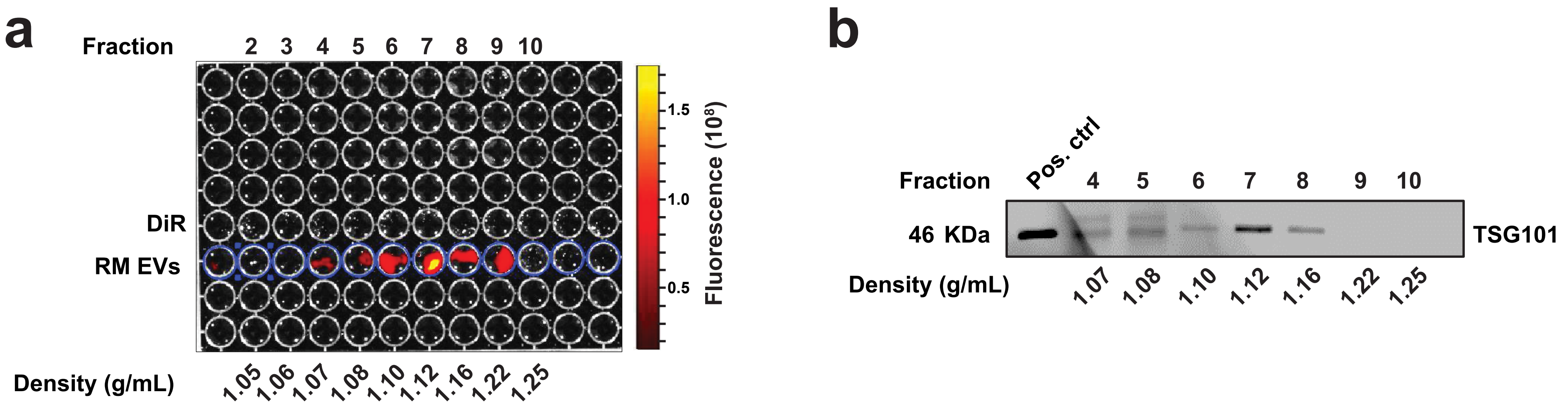

Supplementary Figure 5

Biodistribution of milk derived EVs

a, Image depicting fluorescence of 12 fractions of DiR-labelled EVs and free dye in black 96-well plate after OptiPrep density gradient centrifugation. **b,** Western blot analysis of TSG101 in fractions of DiR-labelled milk derived EVs in increasing density obtained from the OptiPrep density gradient centrifugation.

Supplementary Figure 6

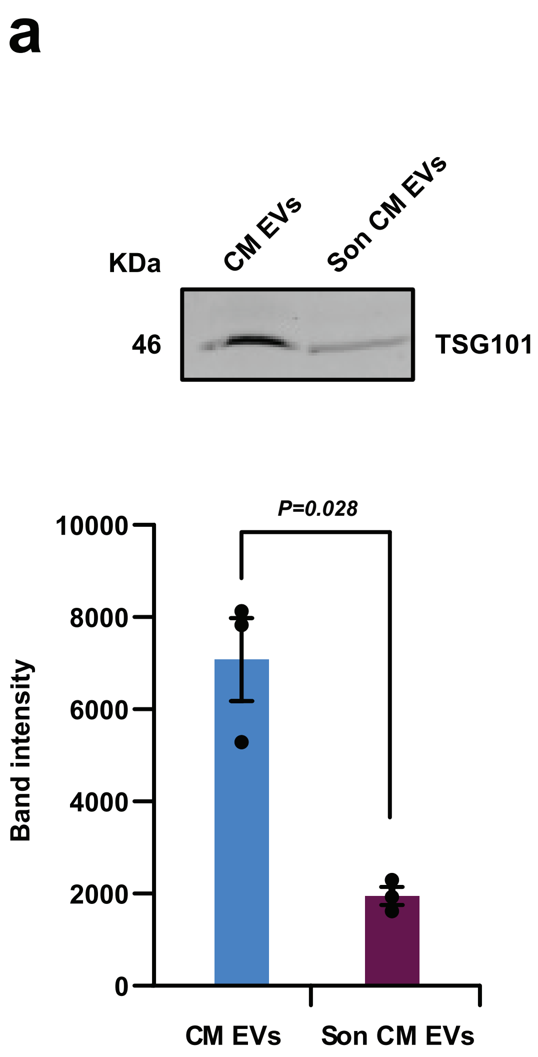

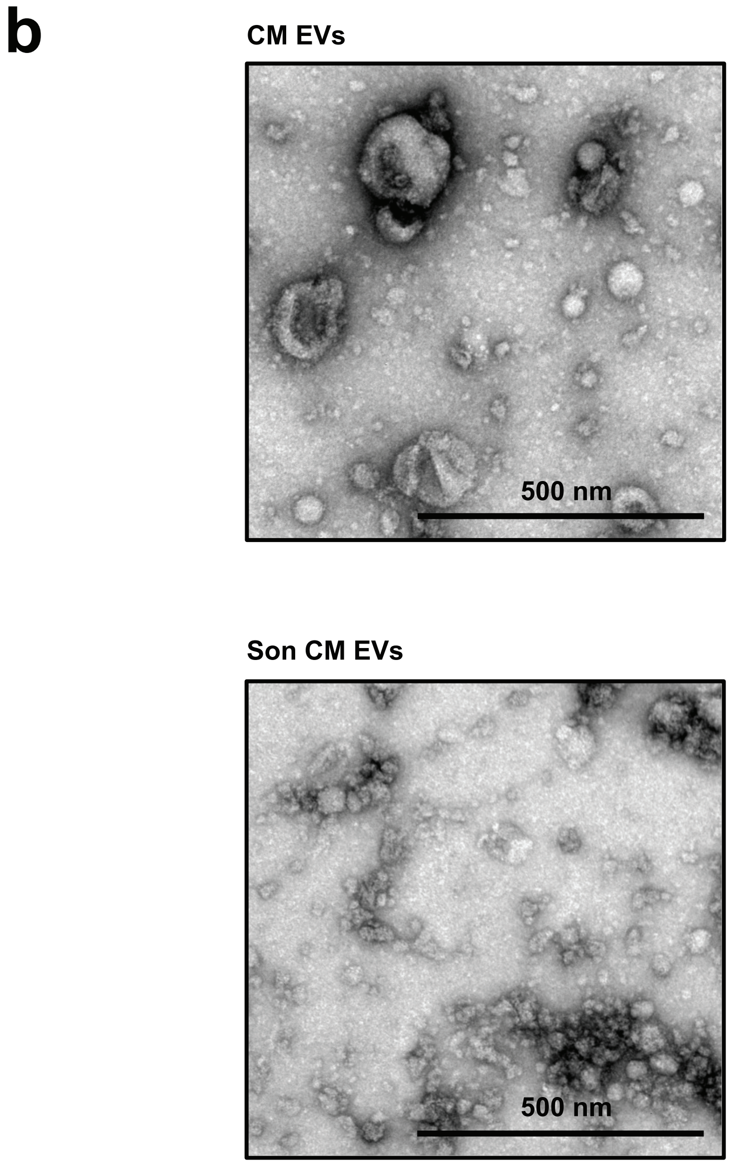

Supplementary Figure 6

Sonication of milk-derived EVs reduces the presence of larger vesicles

a, Western blot analysis confirms that EV enriched luminal protein TSG101 was significantly decreased on sonication of milk-derived EVs (*n*=*3*). All data are represented as mean ± s.e.m. Statistical significance was determined by unpaired two-tailed t-test. **b,** TEM images of sonicated milk-derived EVs show reduction in larger vesicles as compared to the intact CM EVs.

Supplementary Figure 7

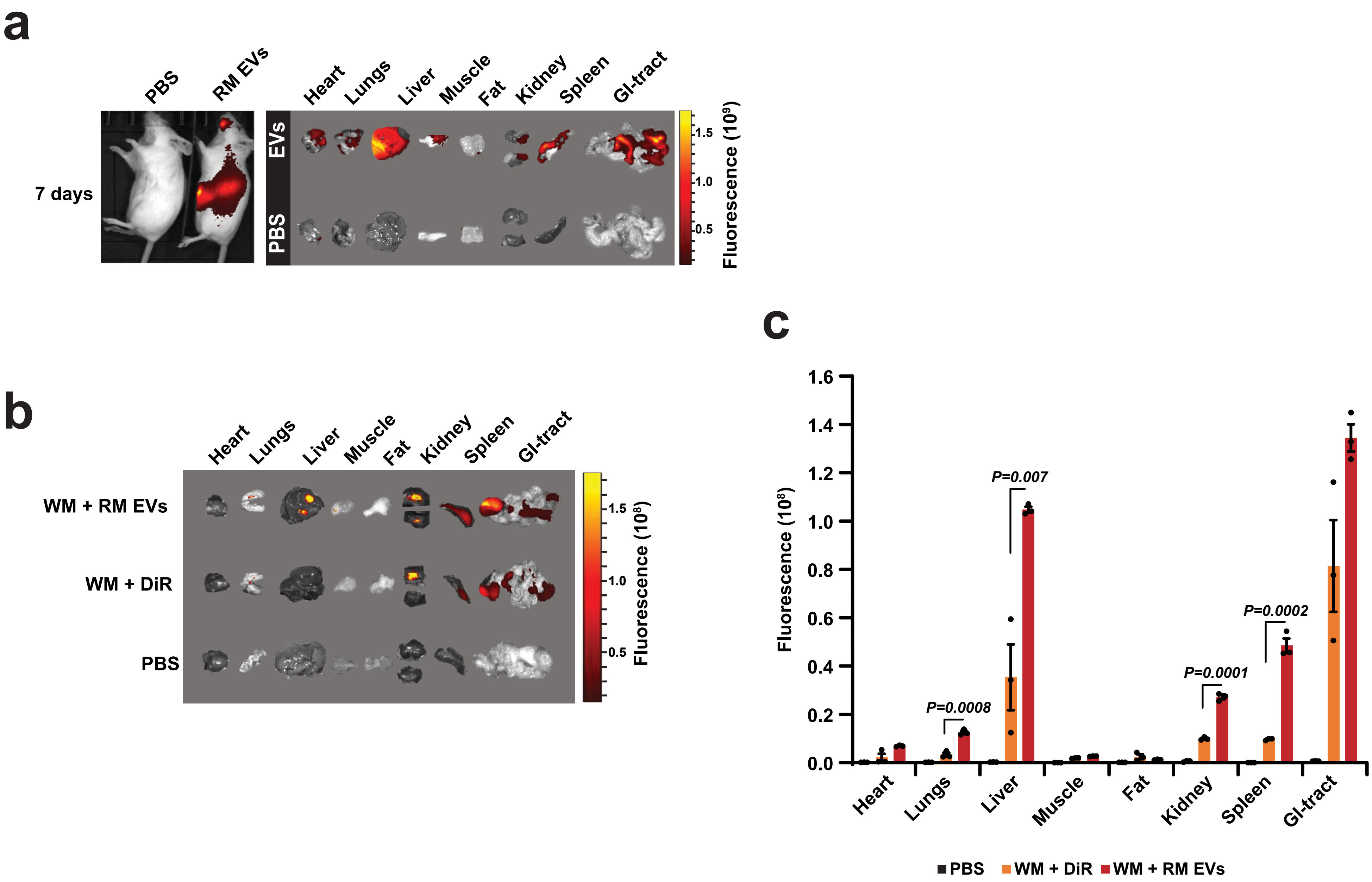

Supplementary Figure 7

Biodistribution of milk derived EVs and whole milk

a, In vivo imaging of mice after 7 days of daily oral administration (p.o.) of DiR labelled milk-derived EVs (25 mg/kg). **b, c,** Female BALB/c mice were administered a single dose of whole CM (60 µL) which was supplemented with 500 µg of DiR-labelled EVs and was compared to whole milk with free dye by *in vivo* imaging after oral administration to the mice (*n=3*). All data are represented as mean ± s.e.m. Statistical significance was determined by unpaired two-tailed t-test.

Supplementary Figure 8

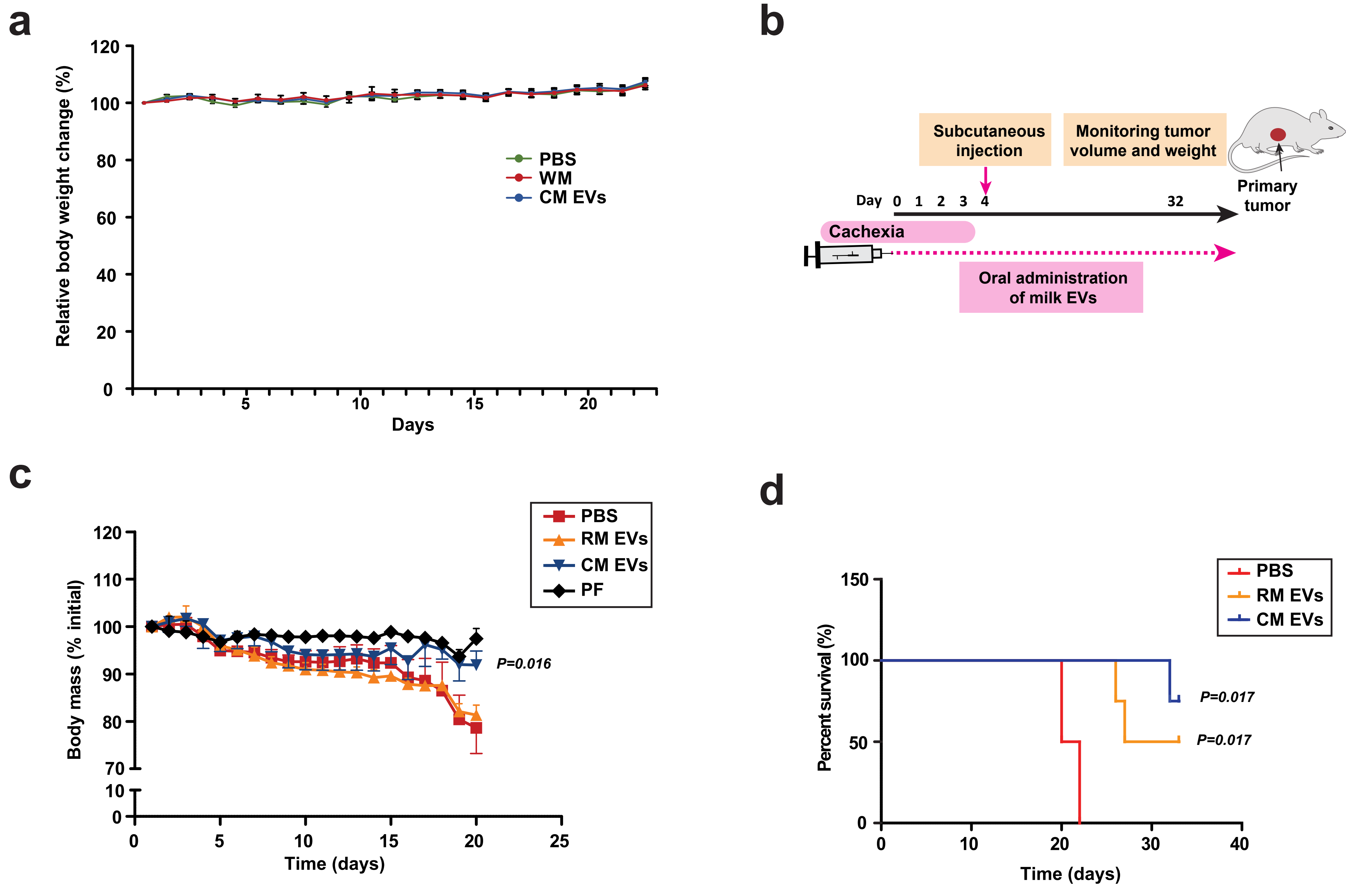

Supplementary Figure 8

Milk-derived EVs attenuates cancer induced weight loss

a, Relative body weight of mice orally administered with PBS, WM (140 µL) and CM EVs (50 mg/kg) (*n*=5). **b,** Schematic representation of cancer cachexia experiment. **c**, Group average mouse weight standardized to starting weight. **d,** Kaplan–Meier curve depicting the survival of immunocompetent C-26 colon cancer mice orally administered with RM and CM EVs (25 mg/kg; *n*=4). PF=pair fed; all data are represented as mean ± s.e.m. Statistical significance was determined by unpaired two-tailed t-test.

Supplementary Figure 9

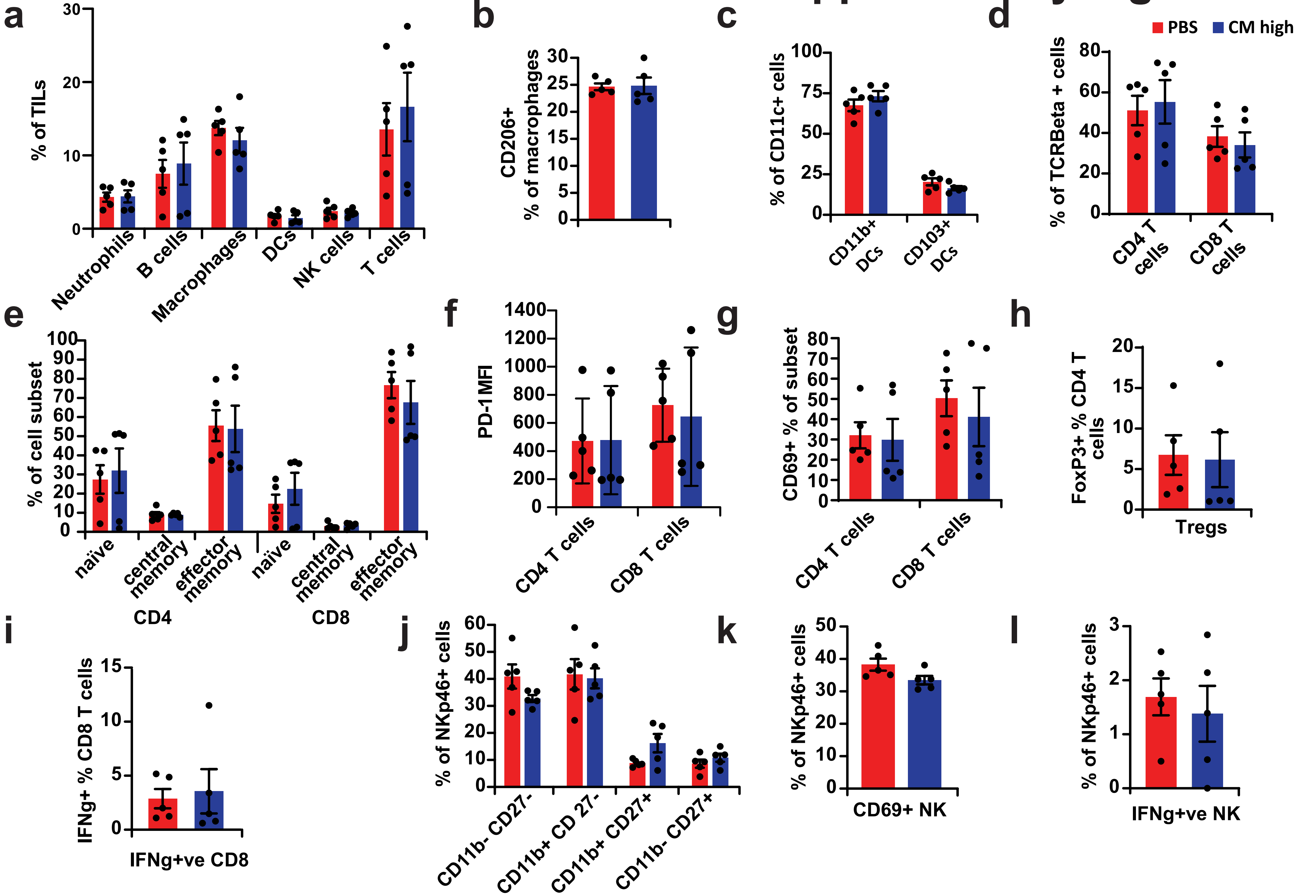

Supplementary Figure 9

Milk EVs do not alter the activation or differentiation of tumor-associated innate and adaptive cells

Primary tumors from PBS control or CM high EV-treated mice were digested and various cell subsets were enumerated by flow cytometry: **a,** Neutrophils: Ly6G^{hi}, Ly6C^{mid}; B cells: CD45R^{+}; Macrophages: F4/80^{+}; Dendritic cells: CD11c^{+}, F4/80^{-}; NK cells: NKp46^{+}, TCRβ^{-}; T cells: TCRβ^{+}, NKp46^{-}. **b,** Tumor-associated macrophages: F4/80^{+}, CD206^{+}, MHC Class II^{hi}. **c,** CD11c^{+} dendritic cells were further segregated into CD11b^{+} and CD103^{+} subsets. T cells were further segregated into **d,** CD4 and CD8 subsets, **e,** CD44 and CD62L differentiation status, activation status by **f,** PD-1 expression and **g,** CD69 expression. **h,** regulatory CD4 T cell numbers and **i,** proportion of tumor-specific CD8 T cells denoted by production of IFNγ. NK cells were segregated into **j,** CD11b and CD27 differentiation status, **k,** activation status denoted by CD69 expression and **l,** tumor-specificity denoted by IFNγ production upon restimulation with tumor cells (*n*=5). All data are represented as mean ± s.e.m. Statistical significance was determined by unpaired two-tailed t-test.

Supplementary Figure 10

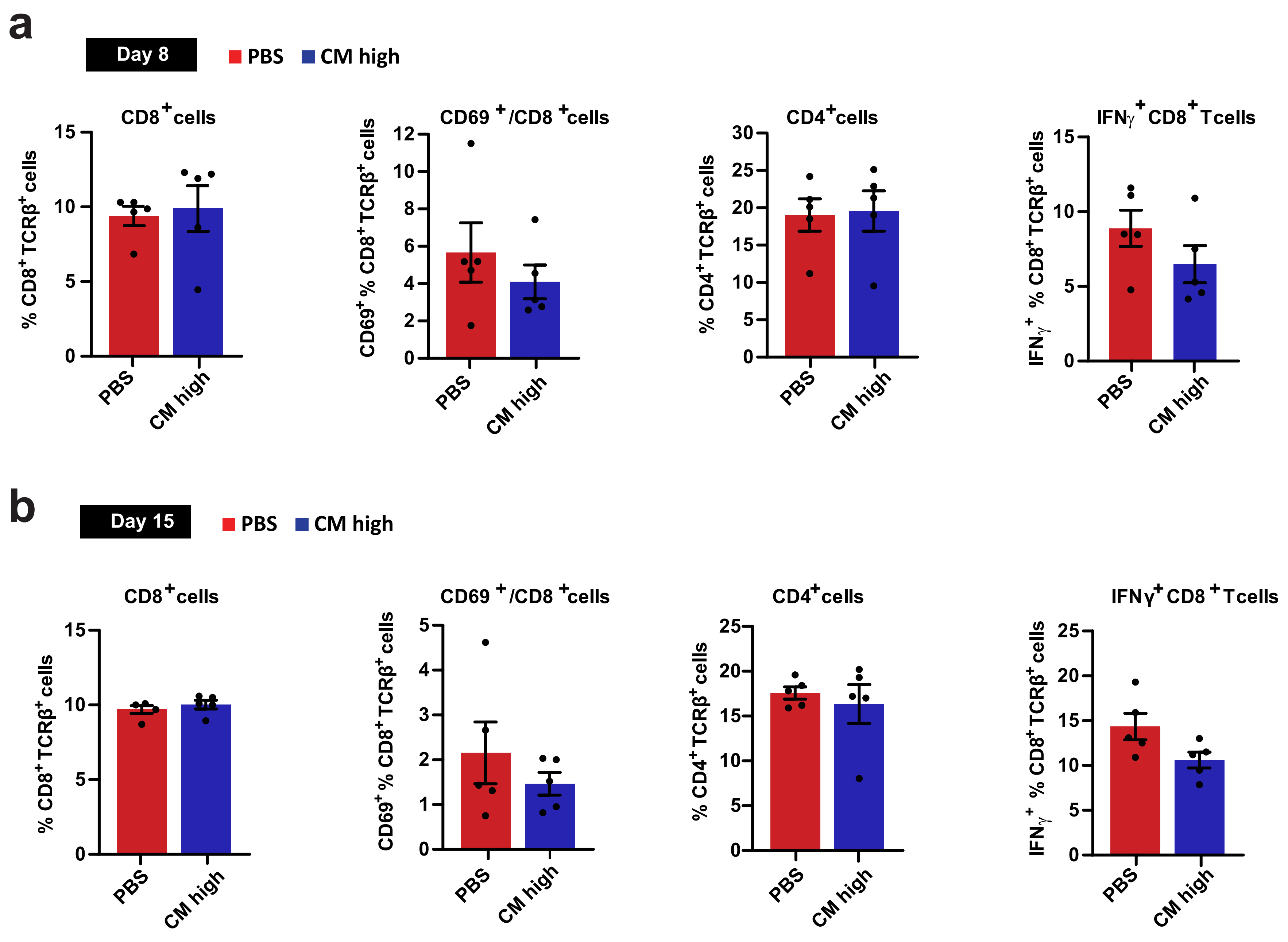

Supplementary Figure 10

Flow cytometric analysis of circulating or infiltrating immune cells in the blood of metastatic 4T1.2 breast cancer mouse model

a, Percentage of circulating $CD4^+$ T-cells, $CD69^+$/$CD8^+$ T-cells, and $CD8^+$ T-cells at day 8 after treatment with PBS and CM EVs. **b,** Percentage of tumor $CD4^+$ T-cells, $CD69^+$/$CD8^+$ T-cells andCD8$^+$ T cells at day 15 after treatment with PBS, and CM EVs (*n*=5). All data are represented as mean ± s.e.m. Statistical significance was determined by unpaired two-tailed t-test.

Supplementary Figure 11

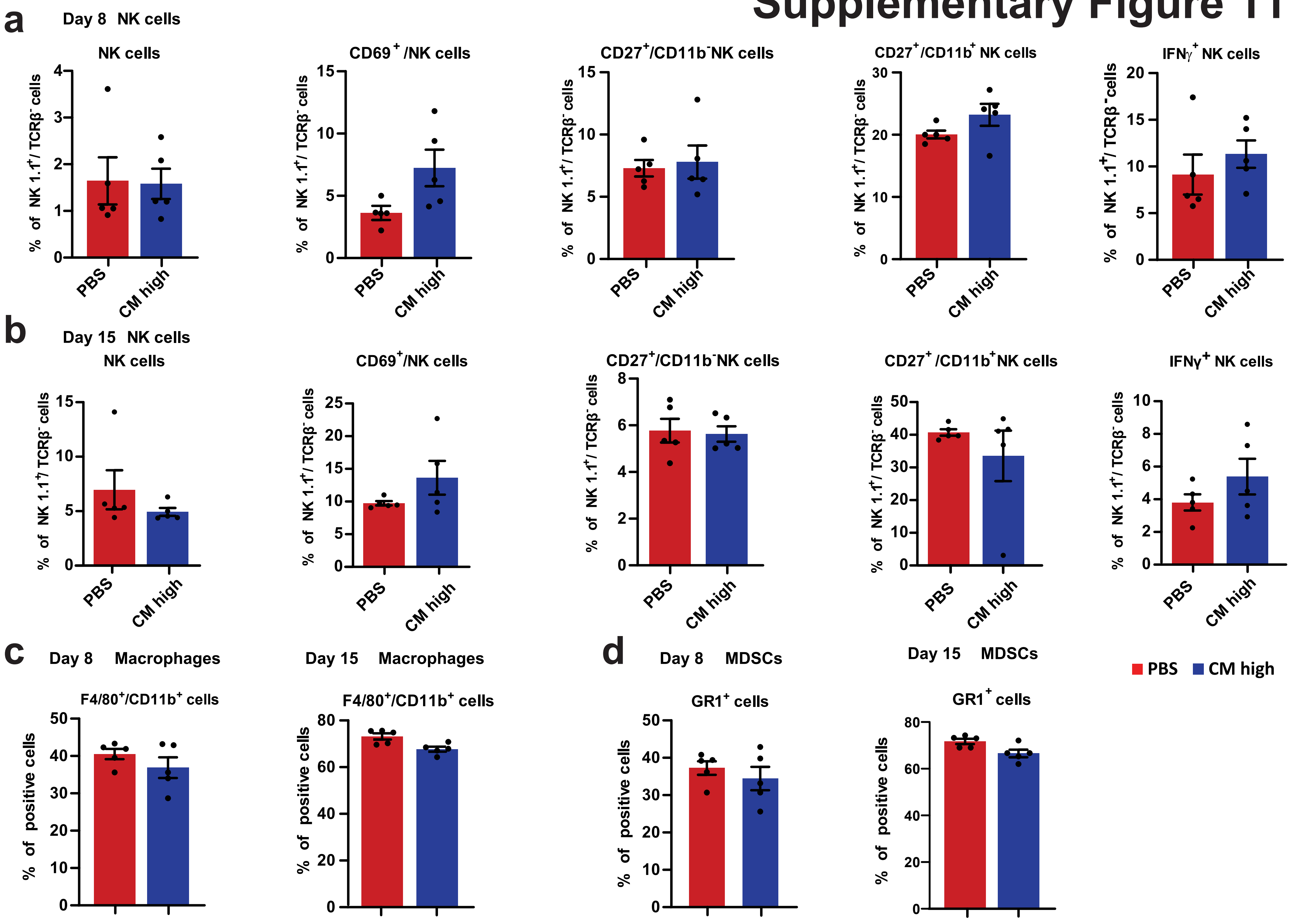

Supplementary Figure 11

Flow cytometric analysis of NK cells, macrophages and MDSCs in the blood of metastatic 4T1.2 breast cancer mouse model

a, Percentage of circulating NK cells at day 8 after treatment with PBS and CM EVs. **b,** Percentage of NK cells at day 15 after treatment with PBS, and CM EVs. **c,** Percentage of macrophages at day 8 and 15 after treatment with PBS and CM EVs. **d,** Percentage of MDSCs at day 8 and 15 after treatment with PBS and CM EVs (n=5). All data are represented as mean ± s.e.m. Statistical significance was determined by unpaired two-tailed t-test.

Supplementary Figure 12

a

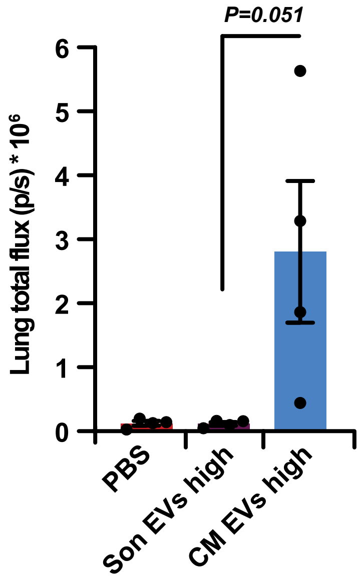

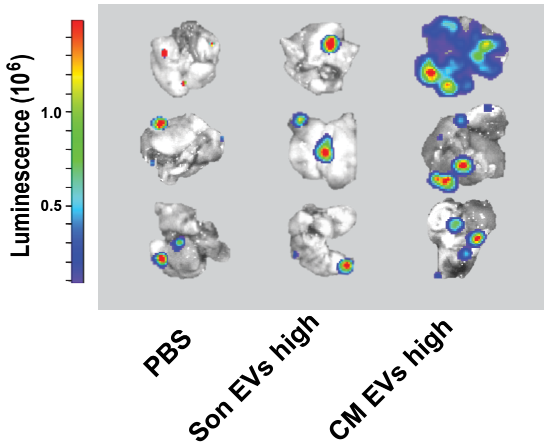

b

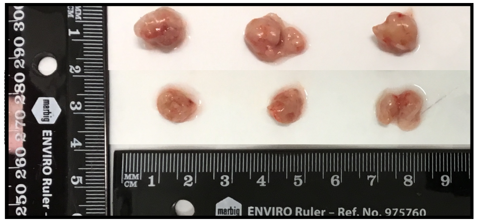

PBS

CM EVs (5 µg)

Supplementary Figure 12

Low amounts of CM EVs reduce primary tumor

a, Quantification of *ex vivo* imaging of lungs from metastatic breast cancer models treated with PBS, sonicated and intact milk-derived CM EVs. Total lung flux to quantify breast cancer metastasis to lungs is depicted (n=5). All data are represented as mean ± s.e.m. Statistical significance was determined by unpaired two-tailed t-test. **b,** Harvested primary tumor size from mice treated with PBS and CM EVs (5 μg) is shown.

Supplementary Figure 13

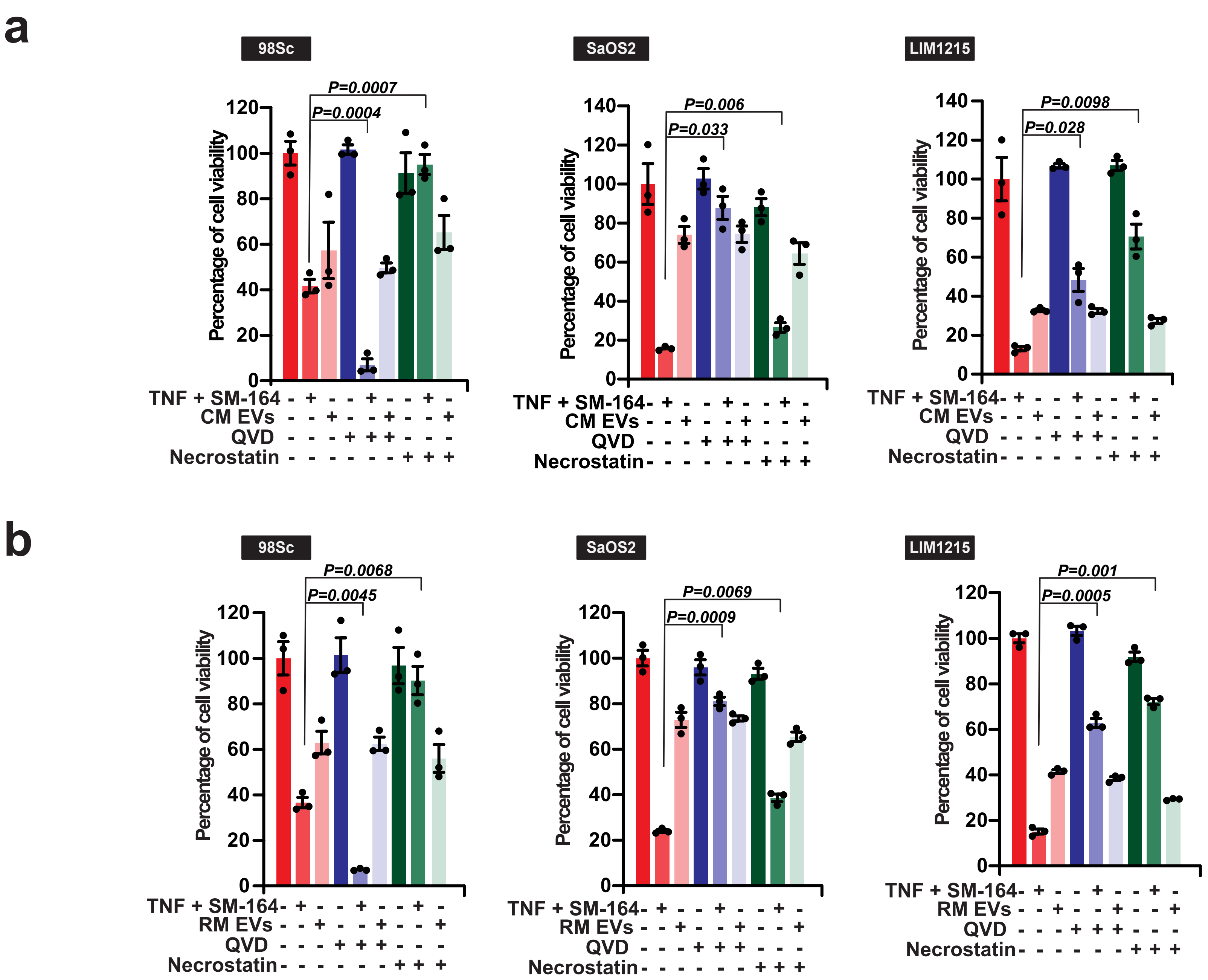

Supplementary Figure 13

Milk-derived EVs reduces the proliferation of cancer cells independent of caspase-dependent apoptosis and necroptosis.

a, Percentage of cell death in cancer cells treated with or without pan-caspase inhibitor QVD or necroptosis inhibitor after treatment with CM EVs. Two osteosarcoma cells and one colorectal cancer (LIM1215) cells were used in this analysis. **b,** Percentage of cell death in cancer cells treated with or without pan-caspase inhibitor QVD or necroptosis inhibitor after treatment with RM EVs (*n*=3). All data are represented as mean ± s.e.m. Statistical significance was determined by two-tailed t-test.

Supplementary Figure 14

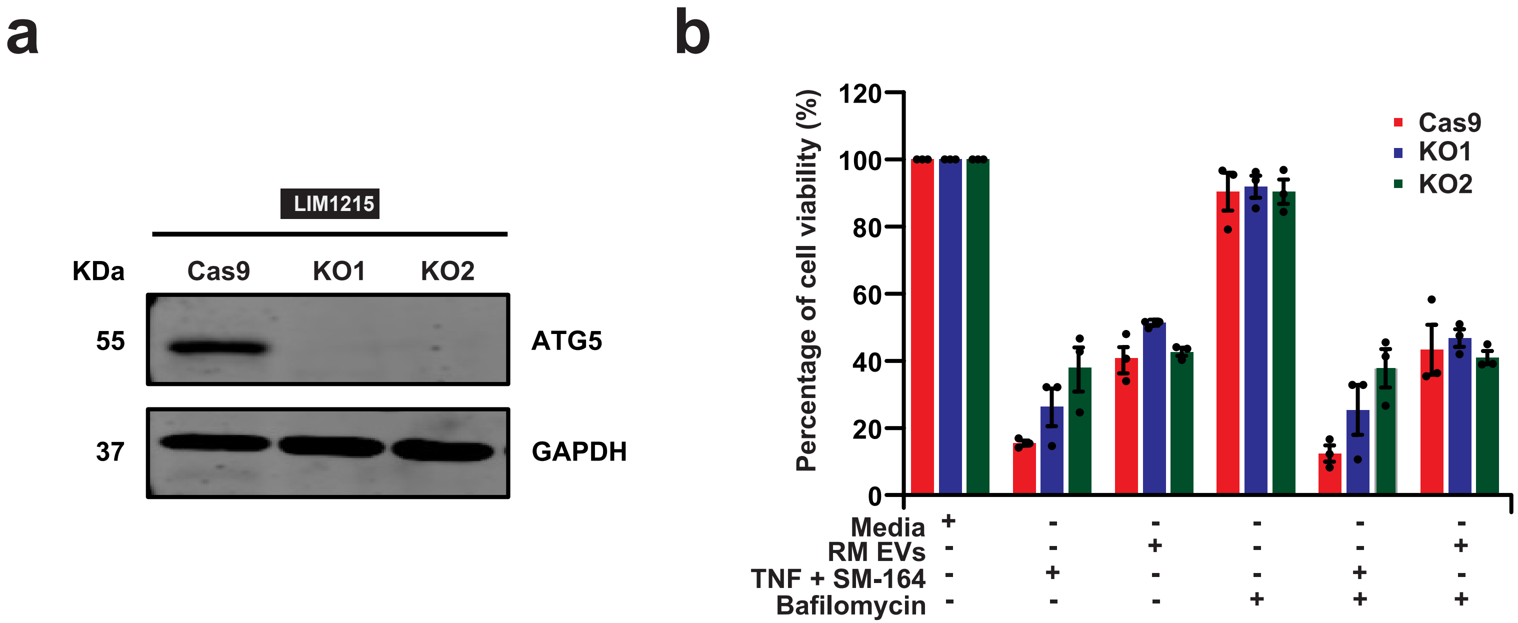

Supplementary Figure 14

Milk-derived EVs does not induce autophagy induced cell death

a, Western blot confirming CRISPR/Cas9 knockout of the autophagy regulator ATG5. **b,** Cell viability of cancer cells did not increase on treatment of $ATG5^{-/-}$ cells with milk-derived EVs (100 µg/mL) or Cas9 cells with the autophagy inhibitor Bafilomycin A (100 nM) (*n*=3). All data are represented as mean ± s.e.m. Statistical significance was determined by two-tailed t-test.

Supplementary Figure 15

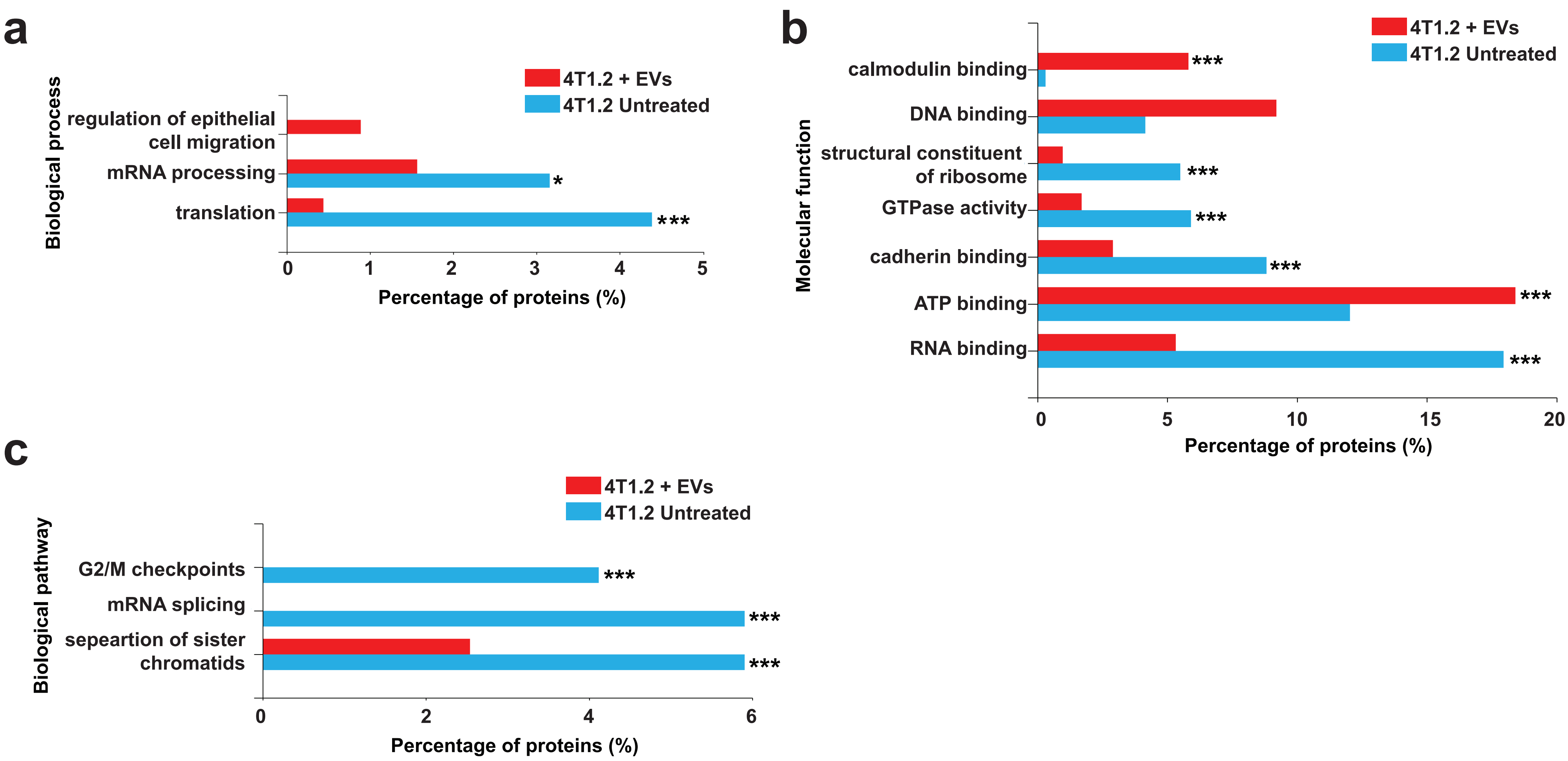

Supplementary Figure 15

Enrichment analysis of proteins dysregulated in cancer cells upon treatment with milk-derived EVs

a, In biological process, proteins implicated in translation and mRNA processing were depleted when 4T1.2 breast cancer cells were treated with milk-derived EVs. On the contrary, proteins implicated in epithelial cell migration were enriched in 4T1.2 cells treated with milk-derived EVs (*$P<0.05$; ***$P<0.001$ as determined by hypergeometric test). **b,** In molecular function, proteins implicated in RNA binding, cadherin binding, GTPase activity and structural constituent of ribosome were depleted in cells treated with milk-derived EVs. Proteins implicated in ATP and calmodulin binding were enriched in cells treated with milk-derived EVs (***$P<0.001$ as determined by hypergeometric test). **c,** In biological pathway, proteins implicated in mRNA splicing, G2/M checkpoints and separation of sister chromatids were depleted in cells treated with milk-derived EVs (***$P<0.001$ as determined by hypergeometric test).

Supplementary Figure 16

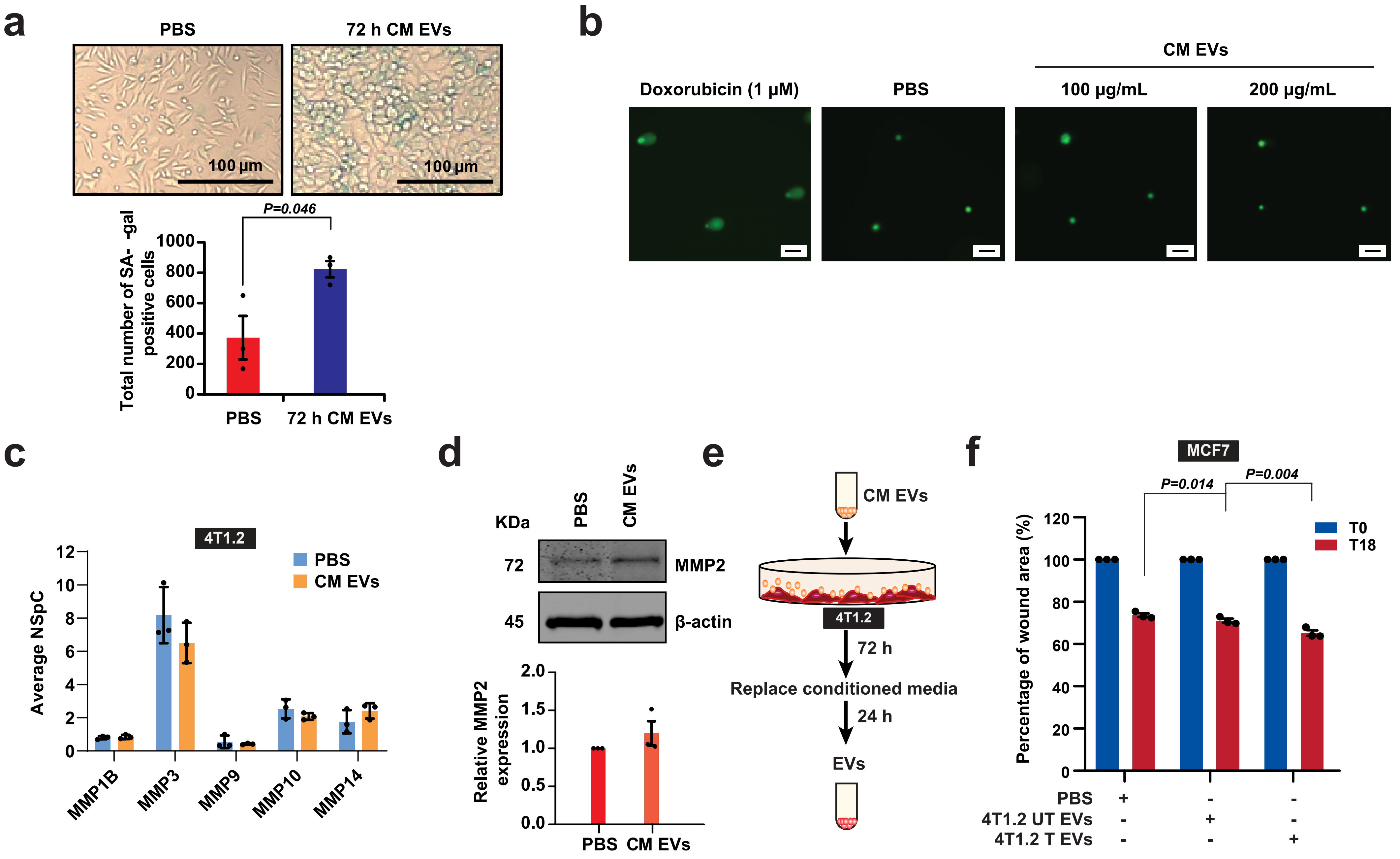

Supplementary Figure 16

Milk-derived EVs induce cellular senescence in cancer cells

a, Total number of senescence-associated β-galactosidase positive cells after treatment with or without milk-derived EVs (100 µg/mL) after 72 h is displayed. **b,** Comet assay depicting DNA damage in 4T1.2 cells in the presence of doxorubicin, PBS and CM EVs. Scale bar represents 100 µM. **c,** Schematic representation of secretome and EVs collection from senescent cells. **d,** Western blotting for MMP2 in secretome of 4T1.2 cells treated with and without CM EVs (100 µg/mL). **e,** Wound healing assay depicts EVs (30 µg/mL) from CM EVs treated cells closed the wound faster. All data are represented as mean ± s.e.m. *n=3*; Statistical significance was determined by two-tailed t-test.

Supplementary Figure 17

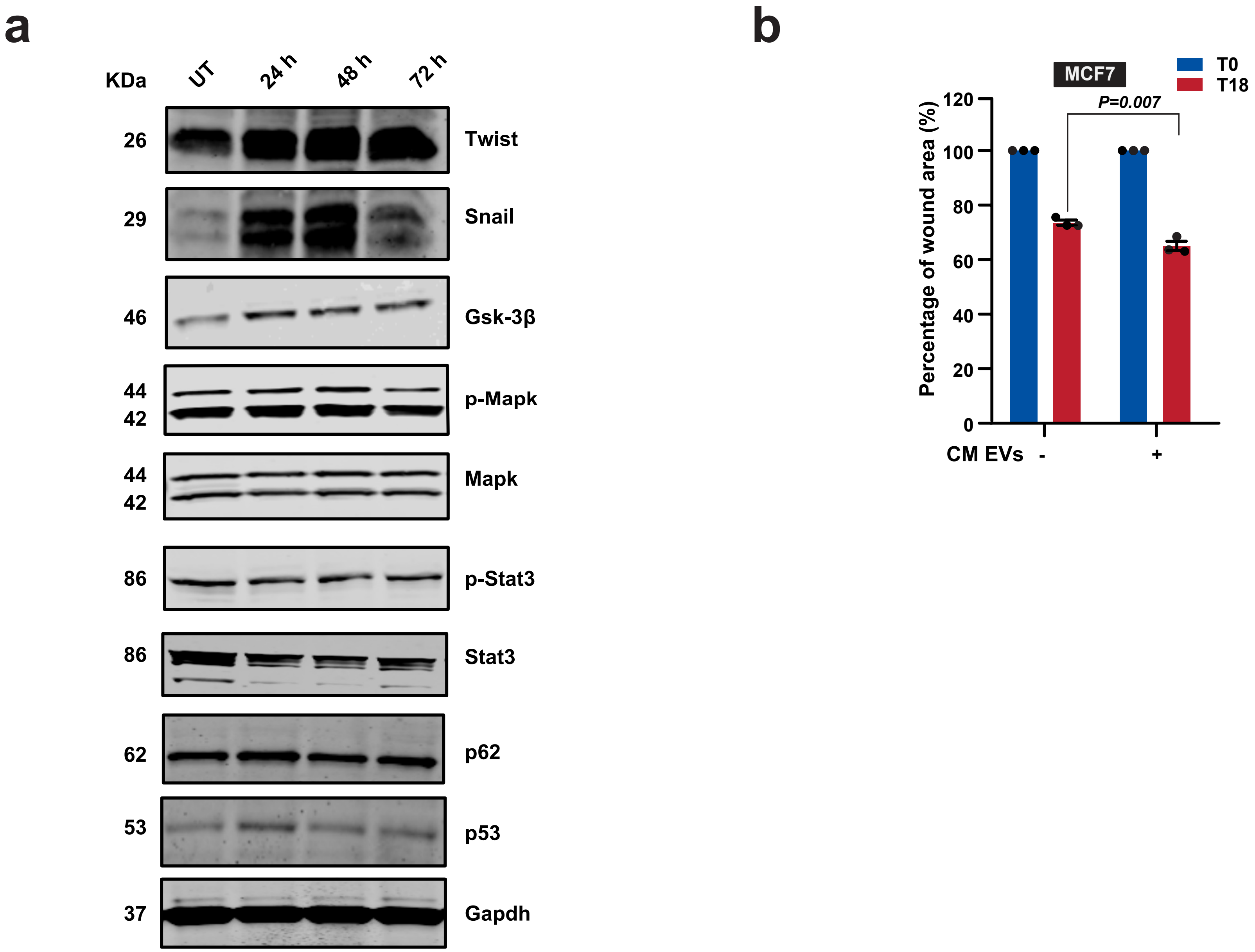

Supplementary Figure 17

Milk-derived EVs induce EMT in cancer cells

a, Western blot analysis of 4T1.2 cancer cells treated with or without milk-derived EVs (100 µg/mL). Molecules implicated in EMT and signalling were probed by Western blotting. **b,** Wound healing assay depicts CM EVs (30 µg/mL) induce significant migration in less invasive MCF7 cells (*n=3*). All data are represented as mean ± s.e.m. Statistical significance was determined by paired two-tailed t-test.

Supplementary Figure 18

a

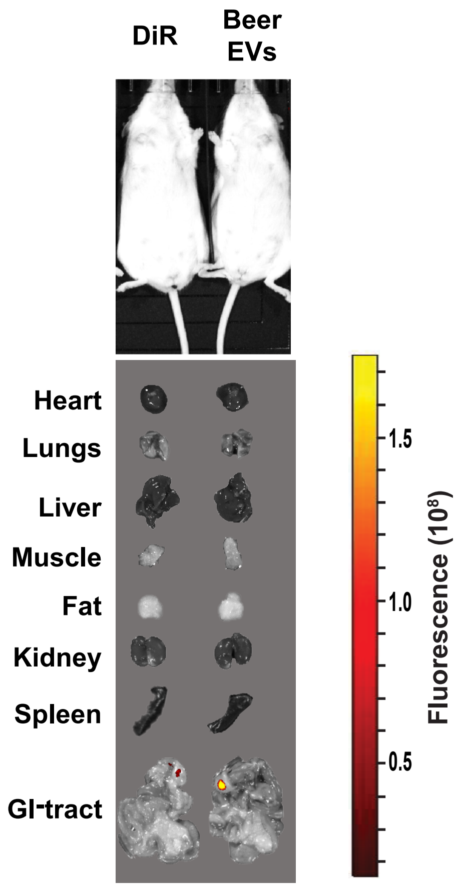

b

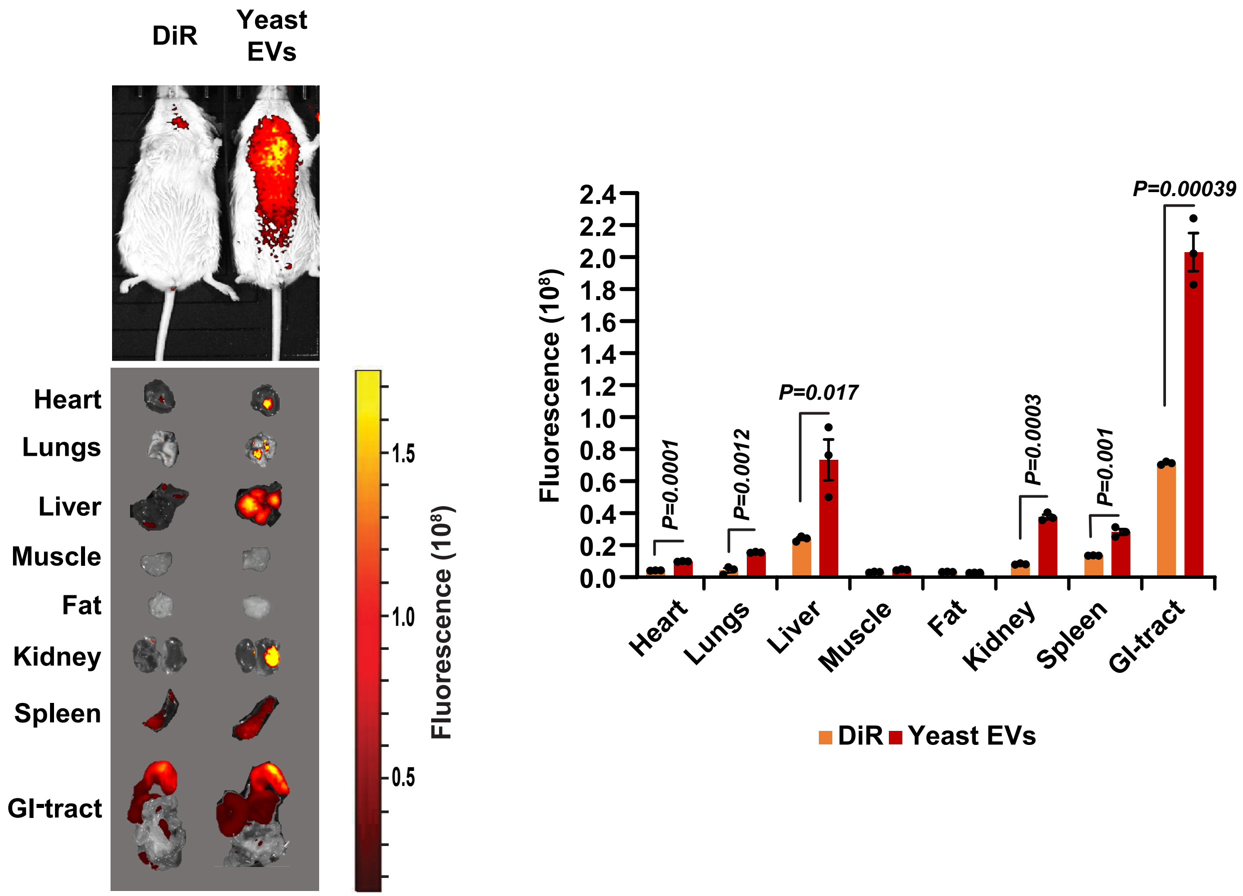

Supplementary Figure 18

Biodistribution of beer and yeast EVs

a, Biodistribution of physiologically relevant concentrations of orally gavaged beer EVs (*n=4*). **b,** Biodistribution of orally gavaged yeast EVs (25 mg/Kg) in mice (*n=4*). All data are represented as mean ± s.e.m. Statistical significance was determined by unpaired two-tailed t-test.

Figure 1

FULL BLOTS

a

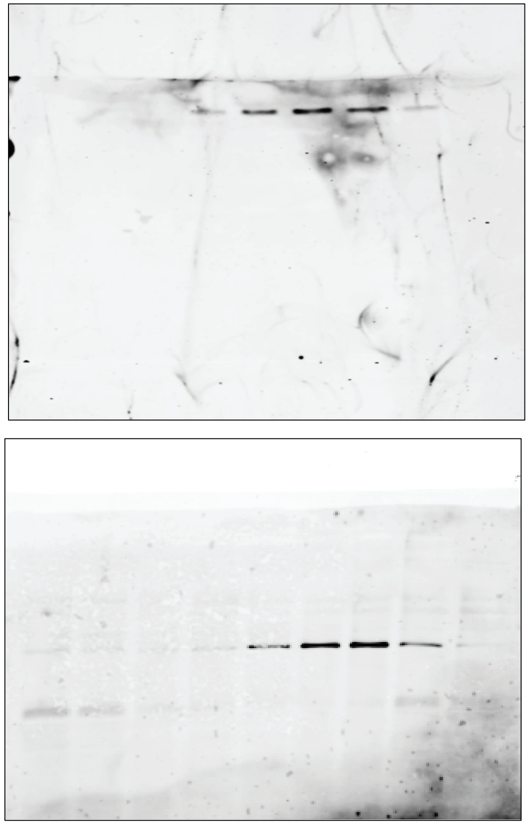

TSG101

TSG101

d

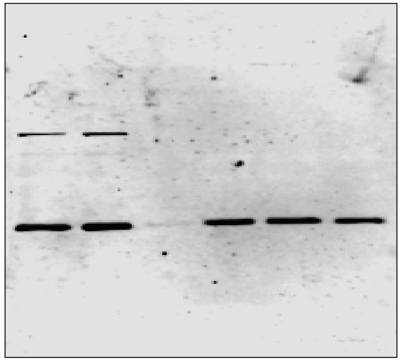

TSG101

f

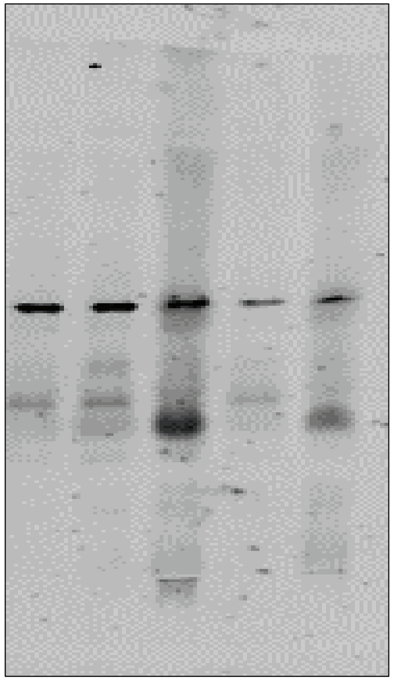

TSG101

Figure 6

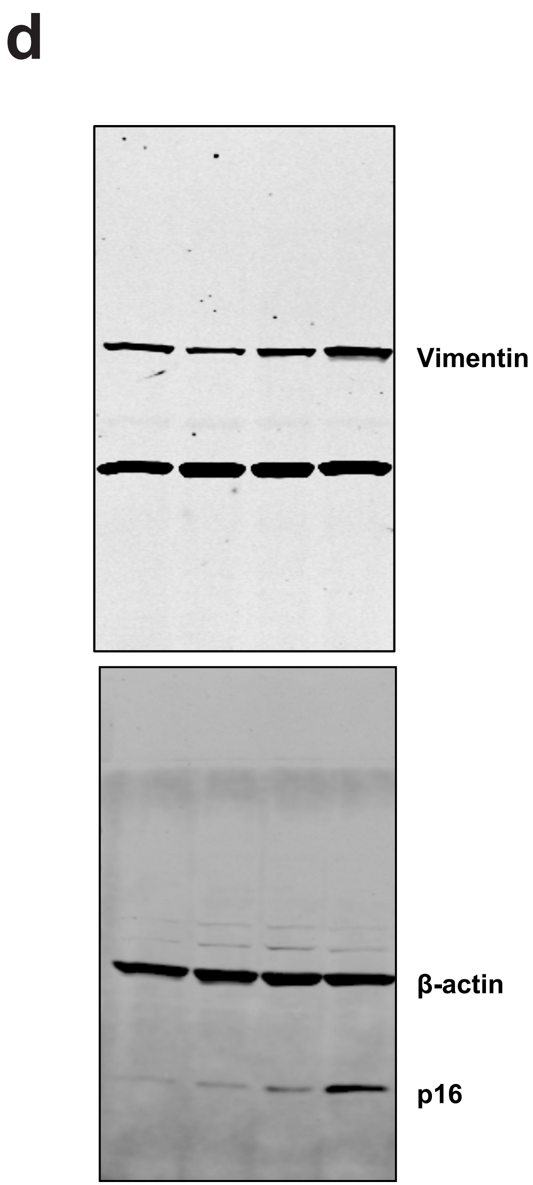

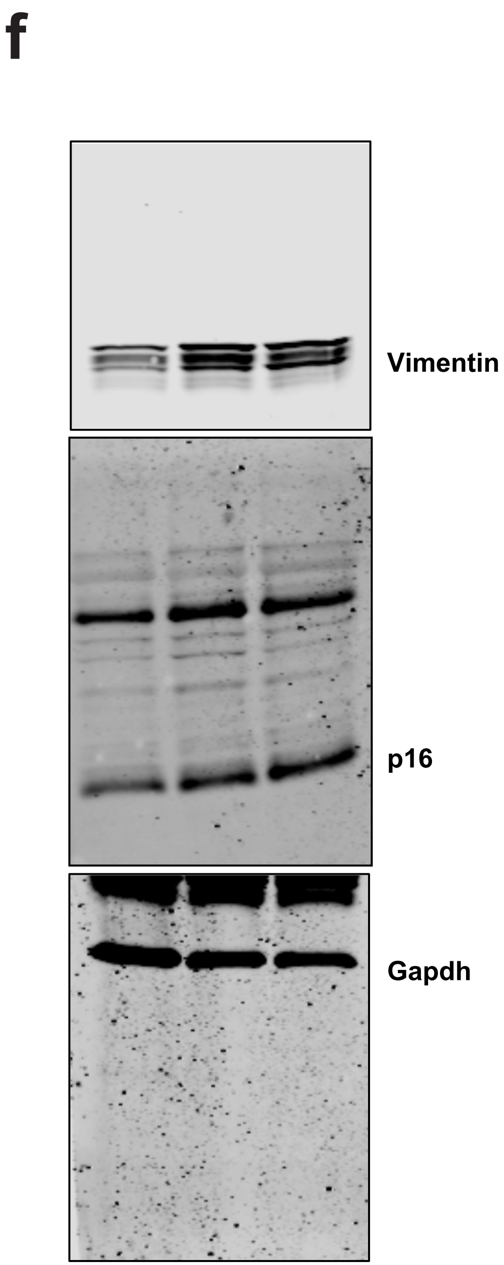

Supplementary Figure 1

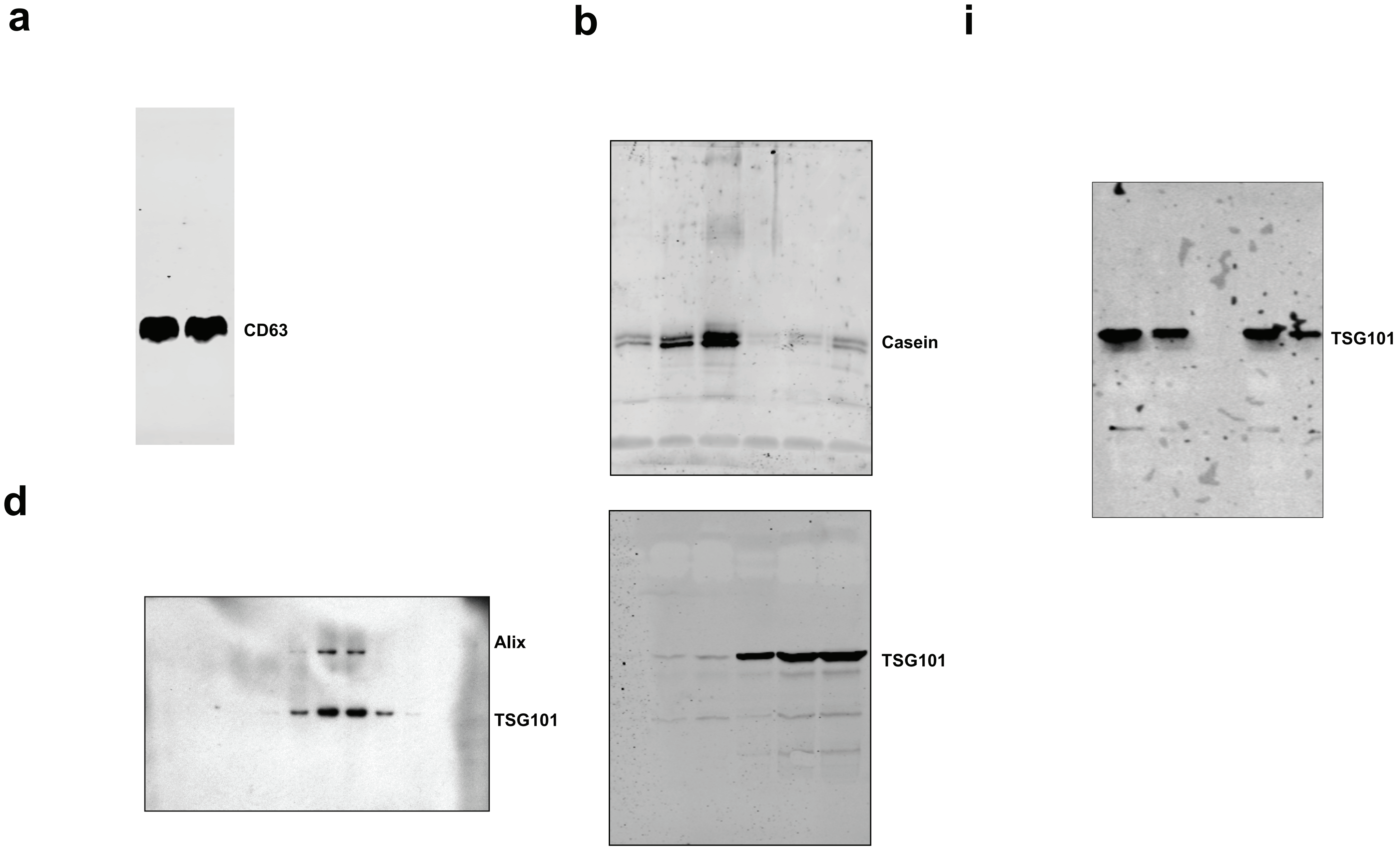

Supplementary Figure 5

b

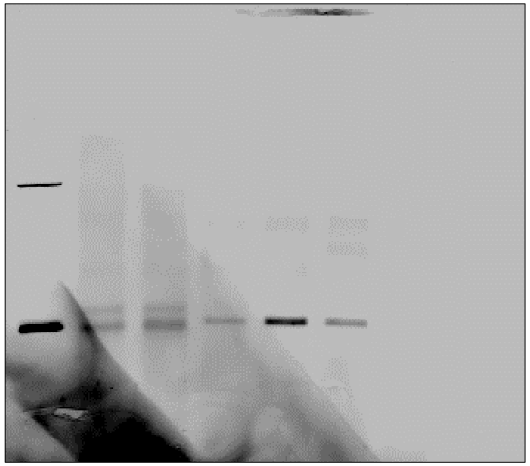

TSG101

Supplementary Figure 6

a

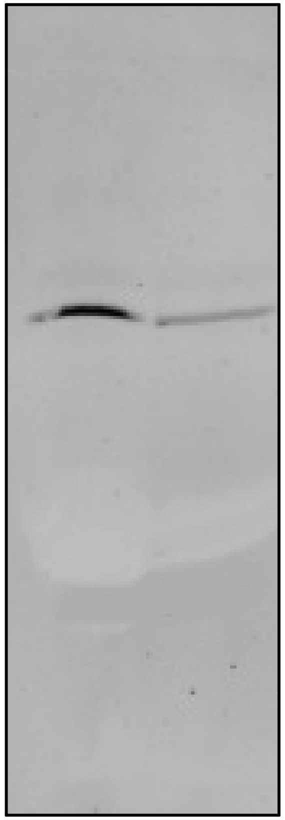

Supplementary Figure 14

a

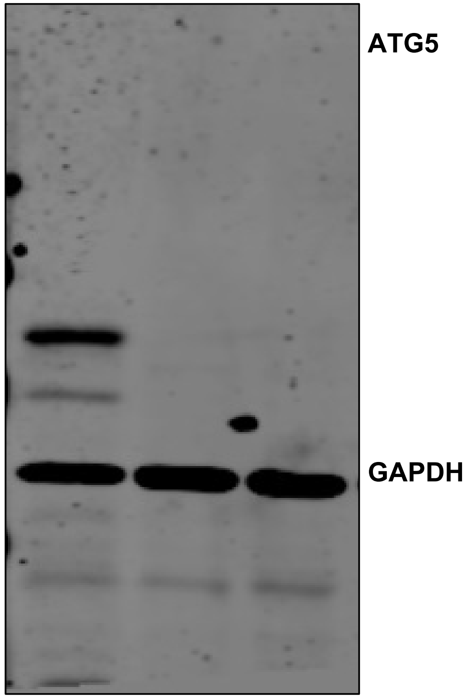

Supplementary Figure 16

d

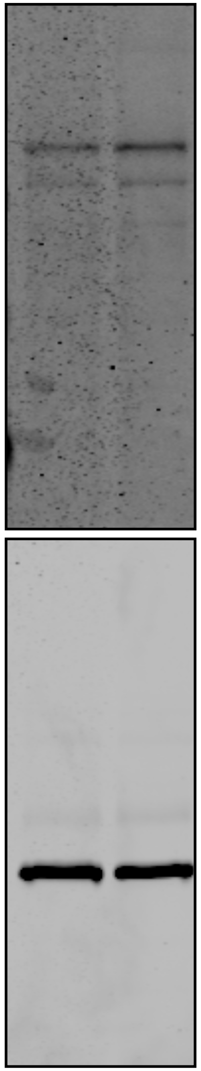

Supplementary Figure 17

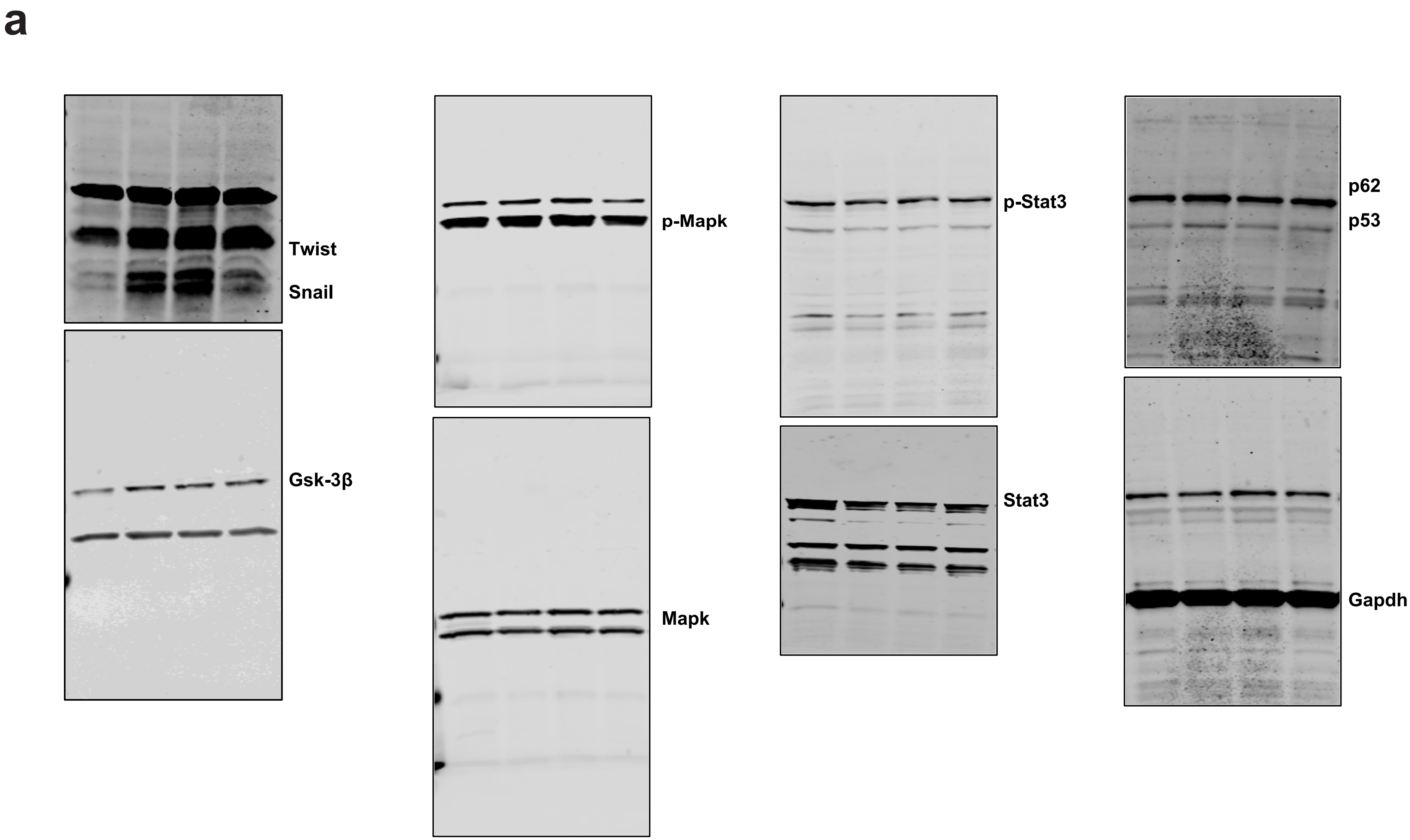

Supplement: Supplementary file 1 — Supplementary information [file 41467_2021_24273_MOESM1_ESM.pdf]
