# Supplementary material for: Oral administration of bovine milk-derived extracellular vesicles induces senescence in the primary tumor but accelerates cancer metastasis
Source: Nat Commun. 2021 Jun 24;12:3950. doi: 10.1038/s41467-021-24273-8 (PMC8225634; doi:10.1038/s41467-021-24273-8)
Supplement: Supplementary file 2 — Description of Additional Supplementary Files [file 41467_2021_24273_MOESM2_ESM.docx]

**Description of Additional Supplementary Files**

**File Name: Supplementary Data 1**

**Description:** List of proteins identified in bovine milk-derived extracellular vesicles

**File Name: Supplementary Data 2**

**Description:** List of RNA identified in bovine milk-derived extracellular vesicles

**File Name: Supplementary Data 3**

**Description:** List of bovine proteotypic tryptic peptides

**File Name: Supplementary Data 4**

**Description:** List of proteins differentially abundant in liver tissue of control and milk EVs treated mice

**File Name: Supplementary Data 5**

**Description:** List of proteins identified in breast cancer cells treated with and without milk EVs for 72 h
