# Supplementary material for: Oral administration of bovine milk-derived extracellular vesicles induces senescence in the primary tumor but accelerates cancer metastasis
Source: Nat Commun. 2021 Jun 24;12:3950. doi: 10.1038/s41467-021-24273-8 (PMC8225634; doi:10.1038/s41467-021-24273-8)
Supplement: Supplementary file 8 — Reporting Summary [file 41467_2021_24273_MOESM8_ESM.pdf]

nature research

Corresponding author(s): Suresh Mathivanan

Last updated by author(s): Mar 18, 2021

Reporting Summary

Nature Research wishes to improve the reproducibility of the work that we publish. This form provides structure for consistency and transparency in reporting. For further information on Nature Research policies, see our Editorial Policies and the Editorial Policy Checklist.

Statistics

For all statistical analyses, confirm that the following items are present in the figure legend, table legend, main text, or Methods section.

n/a	Confirmed	
☐	☒	The exact sample size (n) for each experimental group/condition, given as a discrete number and unit of measurement
☐	☒	A statement on whether measurements were taken from distinct samples or whether the same sample was measured repeatedly
☐	☒	The statistical test(s) used AND whether they are one- or two-sided<br>*Only common tests should be described solely by name; describe more complex techniques in the Methods section.*
☒	☐	A description of all covariates tested
☒	☐	A description of any assumptions or corrections, such as tests of normality and adjustment for multiple comparisons
☐	☒	A full description of the statistical parameters including central tendency (e.g. means) or other basic estimates (e.g. regression coefficient) AND variation (e.g. standard deviation) or associated estimates of uncertainty (e.g. confidence intervals)
☐	☒	For null hypothesis testing, the test statistic (e.g. F, t, r) with confidence intervals, effect sizes, degrees of freedom and P value noted<br>*Give P values as exact values whenever suitable.*
☒	☐	For Bayesian analysis, information on the choice of priors and Markov chain Monte Carlo settings
☒	☐	For hierarchical and complex designs, identification of the appropriate level for tests and full reporting of outcomes
☒	☐	Estimates of effect sizes (e.g. Cohen's d, Pearson's r), indicating how they were calculated

Our web collection on statistics for biologists contains articles on many of the points above.

Software and code

Policy information about availability of computer code

Data collection: Nanosight NTA Version 3.2

Data analysis:
FunRich Version 3.1.3
EV analysis - Nanosight NTA Version 3.2
Data - Microsoft Excel Version 2008
Statistics - GraphPad Prism Version 8.4.2
Western blot - Odyssey image studios Version 3.1
Protein gel - ImageQuant TL Version 8.1
Image J Version 1.47f
FlowJo Version 7.6.2

For manuscripts utilizing custom algorithms or software that are central to the research but not yet described in published literature, software must be made available to editors and reviewers. We strongly encourage code deposition in a community repository (e.g. GitHub). See the Nature Research guidelines for submitting code & software for further information.

Data

Policy information about availability of data

All manuscripts must include a data availability statement. This statement should provide the following information, where applicable:
- Accession codes, unique identifiers, or web links for publicly available datasets
- A list of figures that have associated raw data
- A description of any restrictions on data availability

Data availability statement included in the manuscript. Further information and requests for data should be directed to and will be fulfilled by the Lead Contact,

Suresh Mathivanan (S.Mathivanan@latrobe.edu.au).

Field-specific reporting

Please select the one below that is the best fit for your research. If you are not sure, read the appropriate sections before making your selection.

- [x] Life sciences
- [ ] Behavioural & social sciences
- [ ] Ecological, evolutionary & environmental sciences

For a reference copy of the document with all sections, see nature.com/documents/nr-reporting-summary-flat.pdf

Life sciences study design

All studies must disclose on these points even when the disclosure is negative.

Sample size	No sample size calculation was performed. All in vitro experiments were performed with at least 3 biological replicates. All in vivo experiments were performed with 3 or more mice per group. It is regarded that 3 biological replicates are sufficient to report a biological phenotype in cell culture conditions.
Data exclusions	No data excluded.
Replication	The experiments were performed at least three times independently or in 3 or more mice. All attempts at replication were successful.
Randomization	Samples were allocated to a group based on treatment with or without milk-derived extracellular vesicles.
Blinding	No blinding was done, but two colleagues performed the experiments independently without being influenced by each other. We normally do not perform blinding experiments for cell culture conditions and the same individual performs the cell culture, treatment and processing. For animal experiments, blinding was not done as the groups either receive or not receive treatment and hence the researchers knew the groups at all times.

Reporting for specific materials, systems and methods

We require information from authors about some types of materials, experimental systems and methods used in many studies. Here, indicate whether each material, system or method listed is relevant to your study. If you are not sure if a list item applies to your research, read the appropriate section before selecting a response.

Materials & experimental systems

n/a	Involved in the study	
[ ]	[x]	Antibodies
[ ]	[x]	Eukaryotic cell lines
[x]	[ ]	Palaeontology and archaeology
[ ]	[x]	Animals and other organisms
[x]	[ ]	Human research participants
[x]	[ ]	Clinical data
[x]	[ ]	Dual use research of concern

Methods

n/a	Involved in the study	
[x]	[ ]	ChIP-seq
[ ]	[x]	Flow cytometry
[x]	[ ]	MRI-based neuroimaging

Antibodies

Antibodies used

TSG101 BD Transduction Laboratories #612696
p16 Cell Signaling Technologies®#2407S, #80772
Vimentin Cell Signaling Technologies® #5741S
APDH Cell Signaling Technologies® #5174S
Twist Abcam #ab50857
Snail Cell Signaling Technologies® #3879S
GSK3-β Gene Tex #GTX111192
P-MAPK Cell Signaling Technologies® #9101S
MAPK Cell Signaling Technologies® #9102S
P-Stat3 Cell Signaling Technologies® #9134S
Stat3 Cell Signaling Technologies® #4904S
p62 Cell Signaling Technologies® #2524S
p53 Cell Signaling Technologies® #5114S
CD63 BIO-RAD #MCA2042GA
Ki67 Abcam #ab15580
CD8a-PE-Cy7 (53-6.7) BD Biosciences #552877
CD4-APC-Cy7 (GK1.5) BD Biosciences #552051

CD69-APC (H1.2F3) BD Biosciences #560689
CD279-PE (J43) BD Biosciences #551892
Ly6G-BV421 (1A8) BD Biosciences #551461
Ly6C-APC (HK1.4) Biolegend #128016
β-actin Cell Signaling Technologies® #4970
Caesin Abcam #ab166596
ATG5 Cell Signaling Technologies® #12994S
Eosin Y solution Sigma Aldrich #HT110332
Ki67 Abcam #ab15580
Vimentin Sigma Aldrich #V6630
MMP2 Cell Signaling Technologies® #13132S

Validation	All antibodies have been used according to manufacturer's instructions. For details of verification, relevant citations or further information see the manufacturer's websites.

Eukaryotic cell lines

Policy information about cell lines

Cell line source(s)	Human breast cancer cell line MCF7 was obtained from ATCC<br>Murine breast cancer cell line 4T1 was gifted by Dr. Belinda Parker (La Trobe University).<br>KPC gifted by P Timpson Lab<br>SW620 Gifted by J Mariadason Lab<br>LIM1215 Gifted by J Mariadason Lab<br>C26 Gifted by N Hoogenraad Lab
Authentication	The cell lines used were not authenticated.
Mycoplasma contamination	The cell lines were negative for mycoplasma contamination.
Commonly misidentified lines<br>(See ICLAC register)	No misidentified cell lines were used in this study.

Animals and other organisms

Policy information about studies involving animals; ARRIVE guidelines recommended for reporting animal research

Laboratory animals	Female Balb/c-fox1nuausb (6-8 weeks old), Animal Resources Centre, WA<br>Female Balb/c (8-12 weeks old), Animal Resources Centre, WA<br>Female CD2F1 (8-10 weeks old), Gifted by N Hoogenraad Lab<br><br>Housing conditions:<br>Light cycle 12:12 (7 am lights on and 7 pm lights off)<br>Temperature 22 C (+/- 2 C)<br>Humidity 40-70%
Wild animals	No wild animals were used in this study
Field-collected samples	No field collected samples were used in the study.
Ethics oversight	La Trobe University Animal Ethics Committee<br>Garvan Animal Ethics Committee

Note that full information on the approval of the study protocol must also be provided in the manuscript.

Flow Cytometry

Plots

Confirm that:

- [ ] The axis labels state the marker and fluorochrome used (e.g. CD4-FITC).
- [ ] The axis scales are clearly visible. Include numbers along axes only for bottom left plot of group (a 'group' is an analysis of identical markers).
- [ ] All plots are contour plots with outliers or pseudocolor plots.
- [x] A numerical value for number of cells or percentage (with statistics) is provided.

Methodology

Sample preparation	Cells were seeded at a density of 5000 cells per well in a 24-well plate in 500 μL RPMI 1640 culture medium and allowed to

adhere for 3 and 4 days at 37C at 5% CO2. Cells were then treated with 20 µg/mL of milk-derived EVs and incubated for 72 h. At this time point, cells were scraped and resuspended to collect 200 µL from each well. The medium containing cells were transferred into a 96-well plate and spun at 300 g for 5 min. The supernatant was discarded and the pellet was resuspended in 200 µL of PI-Hypotonic lysis buffer (0.1% (w/v) sodium citrate, 0.1% Triton X 100 (w/v), 50 µg/mL propidium iodide (Sigma Life Science®) in milliQ and incubated overnight at 4C. Samples were then subjected to FACS CANTO II (BD Biosciences).

Instrument	FACS CANTO II (BD Biosciences)
Software	Flow Jo 7.6.2
Cell population abundance	Purity was determine by staining cells with cell-specific markers
Gating strategy	Initial cell population gating was placed on FSC vs SSC (cell size vs granularity). Then, the population of cells were gated on PE 575/26nm-W(dith) and PE 575/26-A(rea) plot. Doublets were removed and the single cells were gated and displayed as a histogram using PE 575/26nm-A parameter. Next, standard cell cycle analysis was performed and cell cycle phases were gated as SubG1, G1, S and G2M phase.

☐ Tick this box to confirm that a figure exemplifying the gating strategy is provided in the Supplementary Information.
